# Vitamin D, DNA methylation, and breast cancer

**DOI:** 10.1186/s13058-018-0994-y

**Published:** 2018-07-11

**Authors:** Katie M. O’Brien, Dale P. Sandler, Zongli Xu, H. Karimi Kinyamu, Jack A. Taylor, Clarice R. Weinberg

**Affiliations:** 1 Biostatistics and Computational Biology Branch, National Institute of Environmental Health Sciences, National Institutes of Health, Research Triangle Park, NC 27709 USA; 2 Epidemiology Branch, National Institute of Environmental Health Sciences, National Institutes of Health, Research Triangle Park, NC 27709 USA; 3 Chromatin and Gene Expression Section, Epigenetics and Stem Cell Biology Laboratory, National Institute of Environmental Health Sciences, National Institutes of Health, Research Triangle Park, NC 27709 USA

**Keywords:** Breast cancer, Vitamin D, 25-Hydroxyvitamin D, DNA methylation, Epigenome-wide association study

## Abstract

**Background:**

Vitamin D has anticarcinogenic and immune-related properties and may protect against some diseases, including breast cancer. Vitamin D affects gene transcription and may influence DNA methylation.

**Methods:**

We studied the relationships between serum vitamin D, DNA methylation, and breast cancer using a case-cohort sample (1070 cases, 1277 in subcohort) of non-Hispanic white women. For our primary analysis, we used robust linear regression to examine the association between serum 25-hydroxyvitamin D (25(OH)D) and methylation within a random sample of the cohort (“subcohort”). We focused on 198 CpGs in or near seven vitamin D-related genes. For these 198 candidate CpG loci, we also examined how multiplicative interactions between methylation and 25(OH)D were associated with breast cancer risk. This was done using Cox proportional hazards models and the full case-cohort sample. We additionally conducted an exploratory epigenome-wide association study (EWAS) of the association between 25(OH)D and DNA methylation in the subcohort.

**Results:**

Of the CpGs in vitamin D-related genes, cg21201924 (*RXRA*) had the lowest *p* value for association with 25(OH)D (*p* = 0.0004). Twenty-two other candidate CpGs were associated with 25(OH)D (*p* < 0.05; *RXRA*, *NADSYN1/DHCR7*, *GC*, or *CYP27B1*). We observed an interaction between 25(OH)D and methylation at cg21201924 in relation to breast cancer risk (ratio of hazard ratios = 1.22, 95% confidence interval 1.10–1.34; *p* = 7 × 10^−5^), indicating a larger methylation-breast cancer hazard ratio in those with high serum 25(OH)D concentrations. We also observed statistically significant (*p* < 0.05) interactions for six other *RXRA* CpGs and CpGs in *CYP24A1*, *CYP27B1*, *NADSYN1/DHCR7*, and *VDR*. In the EWAS of the subcohort, 25(OH)D was associated (*q* < 0.05) with methylation at cg24350360 (*EPHX1*; *p* = 3.4 × 10^−8^), cg06177555 (*SPN*; *p* = 9.8 × 10^−8^), and cg13243168 (*SMARCD2*; *p* = 2.9 × 10^−7^).

**Conclusions:**

25(OH)D concentrations were associated with DNA methylation of CpGs in several vitamin D-related genes, with potential links to immune function-related genes. Methylation of CpGs in vitamin D-related genes may interact with 25(OH)D to affect the risk of breast cancer.

**Electronic supplementary material:**

The online version of this article (10.1186/s13058-018-0994-y) contains supplementary material, which is available to authorized users.

## Background

Vitamin D may protect against poor health outcomes, including heart disease, diabetes, certain cancers, and overall mortality [1–5]. Its biological properties include regulation of cell proliferation and immune function, as well as increased cell differentiation and apoptosis [6–10]. These mechanisms are controlled by the active metabolite 1,25-dihydroxyvitamin D (1,25(OH)_2_D) and the vitamin D receptor (VDR), often in conjunction with retinoid X receptor alpha (RXRA) [11]. This 1,25(OH)_2_D-VDR-RXRA complex binds to vitamin D response elements that can activate or repress gene transcription [12].

Circulating vitamin D levels could affect DNA methylation via transcriptional regulation or other mechanisms [13]. In mammals, DNA methylation is an epigenetic process by which a methyl group is transferred onto the C5 position of a cytosine, forming 5-methylcytosine. Increased methylation at CpG sites in promoter regions is associated with gene inactivation and transcriptional repression, while increased methylation at CpGs in gene bodies is associated with actively transcribed genes [14, 15]. Examples of other environmental exposures associated with methylation changes include smoking (for both smokers [16] and their offspring [17–19]), as well as body mass index (BMI) [20, 21], alcohol consumption [22], and nutrients such as folate, vitamin B12, and retinoic acid [23–27].

Some empirical evidence supports a link between vitamin D exposure and DNA methylation. Candidate gene approaches have observed that vitamin D is associated with methylation of *CYP24A1* [28, 29], *BMP2* [30], *PTEN* [31], and *DKK1* [32]. Additionally, one epigenome-wide association study (EWAS) conducted among adolescent African-American males identified two sites (cg16317961 (*MAPRE2*) and cg04623955 (*DIO3*)) that were significantly associated with serum levels of the stable precursor to 1,25(OH)_2_D, 25-hydroxyvitamin D (25(OH)D) [33]. However, those findings did not replicate in a subsequent EWAS conducted among Caucasian men, nor did that subsequent EWAS identify any novel associations [34]. Another EWAS observed no noteworthy associations between maternal 25(OH)D levels and methylation in cord blood [35], and an epigenome-wide in-vitro study identified no detectable methylation changes in blood mononuclear cells treated with vitamin D [36]. Several studies of the association between vitamin D and LINE-1 global methylation levels have also been negative [37–39].

To further investigate a possible link between vitamin D and DNA methylation, we studied the relationship between serum 25(OH)D and CpGs in or near seven vitamin D-related genes (*VDR*, *RXRA*, *CYP2R1*, *CYP24A1*, *GC*, *CYP27B1*, and *DHCR7*/*NADSYN1*) using a random sample of women from a large prospective cohort (“subcohort”). Based on our previous finding that serum 25(OH)D was associated with a 21% reduction in the hazard of breast cancer over 5 years of follow-up [3], and other research observations that methylation status can modify the responses of individuals to vitamin D treatment [28, 29], we also examined 25(OH)D-methylation interactions in relation to breast cancer risk. We additionally conducted an EWAS of serum 25(OH)D.

## Methods

### Study sample

The Sister Study is a prospective cohort study of 50,884 US women (2003–2009) [40]. At baseline, participants were 35–74 years old and had a sister who had been diagnosed with breast cancer but who had never had breast cancer themselves. Each completed a computer-assisted telephone interview, with in-home collection of anthropometric measurements and blood samples. Participants remain under active surveillance, with more than 90% responding to their most recent follow-up request through March 2015 (data release 4.1). When possible, we collected medical records from self-reported breast cancer cases (82%). Among those with medical records available, 99% of self-reported diagnoses were confirmed.

Participants for a DNA methylation substudy were previously sampled using a case-cohort design [41, 42]. To minimize genetic variation due to racial heterogeneity, this sample was limited to non-Hispanic white women, including all such women who had available blood samples and a self-reported diagnosis of invasive breast cancer or ductal carcinoma in situ*.* The initial methylation sample included 1542 women who developed incident breast cancer between enrollment and March 2015, and a random sample of 1336 women drawn from the full cohort, 74 of whom developed breast cancer by March 2015.

The participants for our previous analysis of serum 25(OH)D and breast cancer [3] were selected to overlap with the case-cohort sample who had DNA methylation data. However, when looking at methylation and 25(OH)D together, we excluded 429 participants who did not have 25(OH)D measured and 102 participants with quality control-related concerns with regard to their DNA methylation (described below). In the end, we had 1070 cases and 1277 in the subcohort (46 of whom were also cases) who had both DNA methylation and serum 25(OH)D data available. All women provided written informed consent and the study was approved by the institutional review boards of the National Institute of Environmental Health Sciences and the Copernicus Group.

### Serum 25(OH)D assessment

Baseline serum was stored at −80 °C before being analyzed using liquid chromatography-mass spectrometry (LC/MS) at Heartland Assays, Inc. (Ames, IA). The three 25(OH)D metabolites—25(OH)D_3_, 25(OH)D_2_, and 3-epi-25(OH)D_3_—were assessed individually, but we summed their concentrations to estimate total 25(OH)D. We adjusted total 25(OH)D values for batch effects using a random effects model and for season of blood draw using LOESS regression. Further details are provided elsewhere [3].

### Methylation analysis

We assessed DNA methylation at 485,512 CpGs (450 K HumanMethylation Beadchip; Illumina, Inc.) using whole blood samples collected from case-cohort participants. Briefly, we extracted 1 μg genomic DNA from whole blood and conducted bisulfite-conversion using the EZ DNA Methylation Kit (Zymo Research, Orange County, CA). Methylation analysis was carried out at the Center for Inherited Disease Research at Johns Hopkins University (Baltimore, MD). Data processing and quality control assessments were completed using the ‘ENMIX’ package (R v3.2.1) [43], and included correcting fluorescent dye-bias [44], quantile normalization [45], and reduction of background noise. We excluded 102 participants whose sample had > 5% low-quality methylation values, low average bisulfite intensity, or implausible methylation value distributions (final *n* = 1277 in subcohort and 1024 additional cases, as described above, plus 123 duplicate samples). We excluded CpGs if they were Illumina-designed single nucleotide polymorphism (SNP) probes, on the Y chromosome, had > 5% low-quality data, were within 2 base pairs of a common SNP, or had multimodal distributions. This left us with 423,500 CpGs. For each site, we calculated a β value based on each individual’s proportion of unmethylated (U) and methylated (M) sites at a given locus: β = M/(U + M + 100).

As interperson variability can be low at some CpGs, we conducted additional screening to better ensure the reliability of our results. We calculated intraclass correlation coefficients (ICCs) to compare the technical variation (within-subject variability, assessed using duplicate samples) to the biologic variation (between-subject variability) [46]. We observed that, for approximately 66% of CpGs, the ICC was less than 0.5, suggesting that there is little interindividual variability and some of the corresponding observed associations may not reflect true biologic differences. We have flagged these CpGs in our results.

### Candidate gene selection

Candidate genes included *VDR* and *RXRA*, as well as the vitamin D binding protein gene (*GC*), and genes directly involved in vitamin D metabolism (*DHCR7/NADSYN1*, *CYP24A1*, *CYP27B1*, and *CYP2R1*). We selected any CpGs included on the 450 K HumanMethylation Beadchip (Illumina, Inc.) located within 2000 base pairs from the candidate gene’s transcription start and end sites, as defined by University of California Santa Cruz Genome Browser (GRCh37/hg19; RefSeq notation) [47]. We identified 198 eligible CpGs.

### Statistical analysis

#### 25(OH)D and methylation of vitamin D-related genes in the subcohort

We assessed the relationship between serum 25(OH)D (continuous, ng/mL) and methylation (continuous, measured as the logit of β) at each of 198 CpGs in or near vitamin D-related genes using robust linear regression with M-estimation. This analysis was limited to the 1270 individuals in the subcohort who had complete information for the following covariates: age at blood draw (continuous), BMI (continuous; kg/m^2^), current smoking status (dichotomous), and alcohol use (never/former drinker, current drinker < 1 drink/day, or current drinker ≥ 1 drink per day). In addition to these covariates, we also adjusted for cell type proportions (CD8 T cells, CD4 T cells, natural killer cells, B cells, monocytes, or granulocytes versus other) [48].

#### 25(OH)D-methylation interaction and breast cancer risk in the case-cohort

Next, we used the case-cohort sample to examine whether interactions between serum 25(OH)D and methylation of vitamin D-related genes were related to breast cancer incidence. This included an assessment of the relationship between methylation at each of the CpG sites in or near vitamin D-related genes and risk of breast cancer. For both sets of analyses, we used Cox proportional hazards models to account for the case-cohort design [41, 42]. We adjusted for age at blood draw, BMI, smoking status, alcohol use, and cell type proportions, as well as education, current hormone therapy use and type, current hormonal birth control use, menopausal status, usual physical activity, history of osteoporosis, parity, and a BMI-menopausal status interaction term. For these candidate CpG locus analyses, we considered *p* < 0.05 to be statistically significant.

For the interaction analysis, the effect measures of interest were ratios of hazard ratios (RHRs). Here, the numerator of the RHR is the hazard ratio (HR) for the association between methylation (measured as 0.1 increments of logit(β)) and breast cancer among those with 25(OH)D levels > 38.0 ng/mL, and the denominator of the RHR is the HR for the association between methylation and breast cancer among those with 25(OH)D levels ≤ 38.0 ng/mL). Therefore, RHR values > 1.00 correspond to a higher estimated HR for the methylation-breast cancer association among those with 25(OH)D levels > 38.0 ng/mL and values < 1.00 correspond to a higher estimated methylation-breast cancer HR among those with 25(OH)D levels ≤ 38.0 ng/mL. The 25(OH)D cut-point was selected based on previous evidence that 38.0 ng/mL is relevant for predicting breast cancer risk [3]. These models also included all of the baseline covariates listed above for the methylation-breast cancer association analysis.

#### Epigenome-wide association study of 25(OH)D in subcohort or cases

We examined the association between serum 25(OH)D and DNA methylation in the subcohort for all 423,500 CpGs from the 450 K panel that passed quality control checks. Here, we corrected for multiple comparisons by calculating false discovery rate *q* values [49], considering those with *q* < 0.05 to be likely to be true positives.

We next assessed the relationship between 25(OH)D and DNA methylation in an independent sample of participants who developed breast cancer within 5 years of enrollment, who were not part of the subcohort, and had the required covariate information (“cases”; *n* = 1024). Here, our goal was to identify CpGs where the 25(OH)D-methylation association differed by future breast cancer status. We compared the subcohort and case results by plotting the –log_10_ *p* values multiplied by the direction of each tested association. We then calculated critical values for a test of the combined *p* values based on Fisher’s method [50]. CpGs that had combined *p* values below identified thresholds were included in additional interaction analyses using the methods described above.

## Results

Women who developed breast cancer during the 5-year follow-up period were slightly older than those in the subcohort (58.7 years versus 55.7 years) and had lower prediagnosis 25(OH)D levels (32.3 versus 32.7 ng/mL). Cases were more likely to have more than one first-degree relative with breast cancer, to be postmenopausal, to be obese, or to be currently taking hormone therapy (Additional file 1: Table S1).

### 25(OH)D and methylation of vitamin D-related genes in the subcohort

**Fig. 1:**
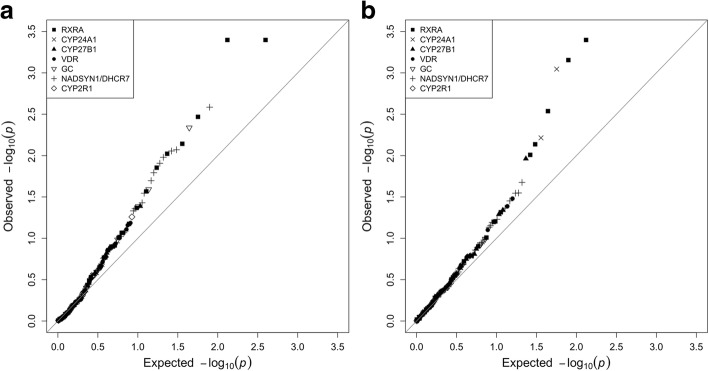
Quantile-quantile plots for vitamin D-related genes. **a** The association between DNA methylation and 25(OH)D in the subcohort. **b** The association between DNA methylation-25(OH)D interactions and breast cancer risk in the case-cohort

Of the 198 CpGs from vitamin D-related genes, cg21201924 (*RXRA*) had the lowest *p* value for association with 25(OH)D in the subcohort (*p* = 0.0004; Table 1 and Additional file 1: Table S2). Twenty-two other candidate CpGs were significantly associated with 25(OH)D, all but one of which were located in *RXRA*, *NADSYN1/DHCR7*, or *GC*. The large overall contrast between our results and those expected by chance is illustrated by a quantile-quantile plot (Fig. 1a).

**Table 1:** CpGs in vitamin D-related genes with statistically significant (*p* < 0.05) associations with 25(OH)D; Sister Study subcohort (*n* = 1270)

Rank	CpG	Gene / location type	Chromosome: position	Mean methylation level (SD)	Association with 25(OH)D^a^	
					β	*p* value
1	cg21201924	RXRA / body	9: 137251825	0.76 (0.042)	−0.020	0.0004
2	cg02127980	RXRA / body	9: 137252116	0.40 (0.067)	−0.015	0.0004
3	cg17559402^b^	NADSYN1 / body	11: 71187890	0.97 (0.006)	−0.017	0.003
4	cg02059519	RXRA / body	9: 137250935	0.83 (0.026)	−0.012	0.003
5	cg09997530	GC / body	4: 72636217	0.91 (0.017)	0.015	0.005
6	cg04329455^b^	RXRA	9: 137215364	0.96 (0.008)	0.014	0.007
7	cg00268518^b^	NADSYN1 / TSS200	11: 71164106	0.01 (0.001)	0.011	0.008
8	cg03146219	NADSYN1 / body	11: 71189514	0.47 (0.097)	−0.012	0.009
9	cg13510651^b^	RXRA / body	9: 137227772	0.94 (0.008)	−0.010	0.01
10	cg03490288^b^	DHCR7 / body	11: 71146658	0.97 (0.006)	0.015	0.01
11	cg05785753	NADSYN1 / body	11: 71189490	0.59 (0.074)	−0.010	0.01
12	cg13687497	RXRA / body	9: 137249839	0.80 (0.023)	−0.010	0.01
13	cg07793224^b^	NADSYN1 / body	11: 71183180	0.97 (0.008)	0.017	0.02
14	cg26044621^b^	DHCR7 / 3´ UTR	11: 71145665	0.94 (0.011)	0.012	002
15	cg04837494	GC / 3´ UTR	4: 72608149	0.85 (0.033)	0.013	0.03
16	cg14236758	RXRA / body	9: 137252129	0.48 (0.066)	−0.009	0.03
17	cg04774822	NADSYN1 / body	11: 71165839	0.78 (0.033)	−0.009	0.03
18	cg16151558^b^	DHCR7 / TSS1500	11: 71159853	0.02 (0.066)	0.013	0.04
19	cg20372759^b^	CYP27B1 / TSS1500	12: 58162287	0.97 (0.006)	−0.010	0.04
20	cg24806812	GC / body	4: 72635202	0.93 (0.018)	0.012	0.04
21	cg14154547^b^	RXRA / body	9: 137293309	0.92 (0.010)	−0.007	0.04
22	cg07099121^b^	DHCR7 / 3´ UTR	11: 71146096	0.98 (0.004)	−0.010	004
23	cg16910670^b^	NADSYN1	11: 71215361	0.97 (0.005)	−0.011	0.05

*TSS200* within 200 basepairs upstream of the transcription start site, *TSS1500* within 1500 basepairs upstream of the transcription start site, *UTR* untranslated region

^a^Estimated change in methylation (logit(β)) per 10 ng/mL change in serum 25-hydroxyvitamin D (25(OH)D)

^b^Intraclass correlation coefficient < 0.5

### 25(OH)D-methylation interaction and breast cancer risk in the case-cohort

Eighteen of the 198 candidate CpGs showed evidence of interacting with 25(OH)D to affect breast cancer risk in the case-cohort sample (*p* < 0.05; Table 2 and Additional file 1: Table S3). This is more than expected by chance, as illustrated by the quantile-quantile plot (Fig. 1b). Nine of the eighteen had ICCs > 0.5. Only one was directly associated with breast cancer risk (cg10592901 in *VDR*, HR = 1.04, 95% confidence interval (CI) 1.01–1.07).

**Table 2:** Interacting effects of 25(OH)D and methylation at CpG sites in vitamin D-related genes on the 5-year risk of breast cancer (1024 cases, 1270 from subcohort, including 46 additional cases^a^): ratio of hazard ratios and 95% confidence intervals for CpGs with statistically significant interactions (*p* < 0.05)

Rank	CpG site	Gene / location type	HR (95% CI) for methylation-breast cancer association	HR (95% CI) for methylation-breast cancer association, if 25(OH)D ≤ 38.0 ng/mL	HR (95% CI) for methylation-breast cancer association, if 25(OH)D > 38.0 ng/mL	Ratio of Hazard Ratios (95% CI)^b^	Interaction *p* value
1	cg21201924	RXRA / body	1.00 (0.96–1.04)	0.97 (0.93–1.01)	1.18 (1.08–1.29)	1.22 (1.10–1.34)	7.0 × 10^−5^
2	cg13786567	RXRA / body	1.01 (0.94–1.08)	0.94 (0.87–1.02)	1.34 (1.12–1.60)	1.42 (1.17–1.73)	4.0 × 10^−4^
3	cg02127980	RXRA / body	1.00 (0.94–1.06)	0.95 (0.88–1.01)	1.22 (1.07–1.38)	1.29 (1.11–1.49)	7.0 × 10^−4^
4	cg12978433	CYP24A1 / 1st exon	1.01 (0.98–1.04)	1.04 (1.00–1.07)	0.93 (0.88–0.98)	0.90 (0.84–0.96)	9.0 × 10^−4^
5	cg14154547^c^	RXRA / body	0.96 (0.90–1.03)	0.91 (0.84–0.98)	1.17 (1.01–1.36)	1.29 (1.09–1.53)	0.003
6	cg18956481^c^	CYP24A1 / 5´ UTR	0.99 (0.97–1.02)	1.01 (0.99–1.04)	0.93 (0.88–0.98)	0.92 (0.86–0.98)	0.006
7	cg13510651^c^	RXRA / body	0.99 (0.93–1.06)	0.95 (0.88–1.02)	1.18 (1.03–1.35)	1.24 (1.06–1.45)	0.007
8	cg14236758	RXRA / body	1.00 (0.94–1.06)	0.96 (0.90–1.03)	1.19 (1.03–1.37)	1.23 (1.05–1.44)	0.01
9	cg09253762	CYP27B1 / TSS1500	0.99 (0.95–1.04)	0.96 (0.91–1.01)	1.12 (1.01–1.24)	1.16 (1.04–1.31)	0.01
10	cg18482822^c^	DHCR7 / body	1.02 (0.97–1.06)	0.99 (0.93–1.04)	1.13 (1.02–1.24)	1.14 (1.02–1.27)	0.02
11	cg05072492^c^	NADSYN1 / TSS1500	1.01 (0.96–1.06)	0.97 (0.92–1.03)	1.11 (1.00–1.24)	1.15 (1.01–1.29)	0.03
12	cg11035813^c^	DHCR7 / TSS1500	1.02 (0.98–1.07)	1.00 (0.95–1.05)	1.13 (1.02–1.24)	1.13 (1.01–1.26)	0.03
13	cg14854850	VDR / 3´ UTR	0.99 (0.94–1.03)	1.02 (0.97–1.07)	0.90 (0.82–0.99)	0.89 (0.79–0.99)	0.03
14	cg25588697^c^	DHCR7 / body	1.01 (0.95–1.08)	0.96 (0.89–1.05)	1.14 (1.00–1.30)	1.18 (1.01–1.38)	0.04
15	cg10592901	VDR / body	1.04 (1.01–1.07)	1.06 (1.03–1.10)	0.98 (0.92–1.05)	0.92 (0.86–1.00)	0.04
16	cg12474705^c^	NADSYN1 / body	0.96 (0.92–1.01)	0.99 (0.94–1.05)	0.88 (0.80–0.98)	0.89 (0.79–1.00)	0.04
17	cg16984335^c^	CYP27B1 / body	1.00 (0.96–1.04)	1.02 (0.97–1.06)	0.92 (0.83–1.00)	0.90 (0.81–1.00)	0.05
18	cg13941235	RXRA / body	1.00 (0.97–1.02)	0.98 (0.95–1.01)	1.04 (0.99–1.10)	1.06 (1.00–1.13)	0.05

*CI* confidence interval, *HR* hazard ratio, *TSS1500* within 1500 basepairs upstream of the transcription start site, *UTR* Untranslated region

^a^After excluding those with missing covariate information

^b^Change in the methylation-breast cancer association for being in the 4th quartile of 25-hydroxyvitamin D (25(OH)D) (≥ 38.0 ng/mL) versus the first three (< 38.0 ng/mL)—a value > 1.00 indicates that the estimated HR for the methylation-breast cancer association is higher among those with higher 25(OH)D levels; similarly, an RHR < 1.00 indicates that the estimated HR for the methylation-breast cancer association is higher among those with lower 25(OH)D levels

^c^Intraclass correlation coefficient < 0.5

The CpG with the smallest *p* value for the 25(OH)D-methylation interaction analysis was cg21201924 (*RXRA*). Among women with 25(OH)D levels > 38.0 ng/mL, each 0.1 change in logit(β) was associated with an 18% increase in the breast cancer hazard (HR = 1.18, 95% CI 1.08–1.29). By contrast, among women with 25(OH)D levels ≤ 38.0 ng/mL, each 0.1 change in logit(β) was associated with a 3% lower hazard of developing breast cancer (HR = 0.97, 95% CI 0.93–1.01). The corresponding RHR was 1.22 (95% CI 1.10–1.29; *p* = 7.0 × 10^−5^).

Six other *RXRA* CpGs (cg13786567, cg02127980, cg14154547, cg13510651, cg14236758, and cg13941235) also showed evidence of interacting with 25(OH)D to affect breast cancer risk. Other statistically significant sites included cg12978433 and cg18956481 in *CYP24A1*, cg09253762 and cg16984335 in *CYP27B1*, cg18482822, cg05072492, cg11035813, cg25588697, and cg12474705 in *NADSYN1*/*DHCR7*, and cg14854850 and cg10592901 in *VDR*. As we have previously reported that the protective association between 25(OH)D and breast cancer appears to be limited to postmenopausal women [3], we present postmenopause-specific analyses in Additional file 1: Tables S4 and S5 and Figure S1.The results were largely consistent with the analyses that included all breast cancers.

### Epigenome-wide association study of 25(OH)D in subcohort or cases

**Fig. 2:**
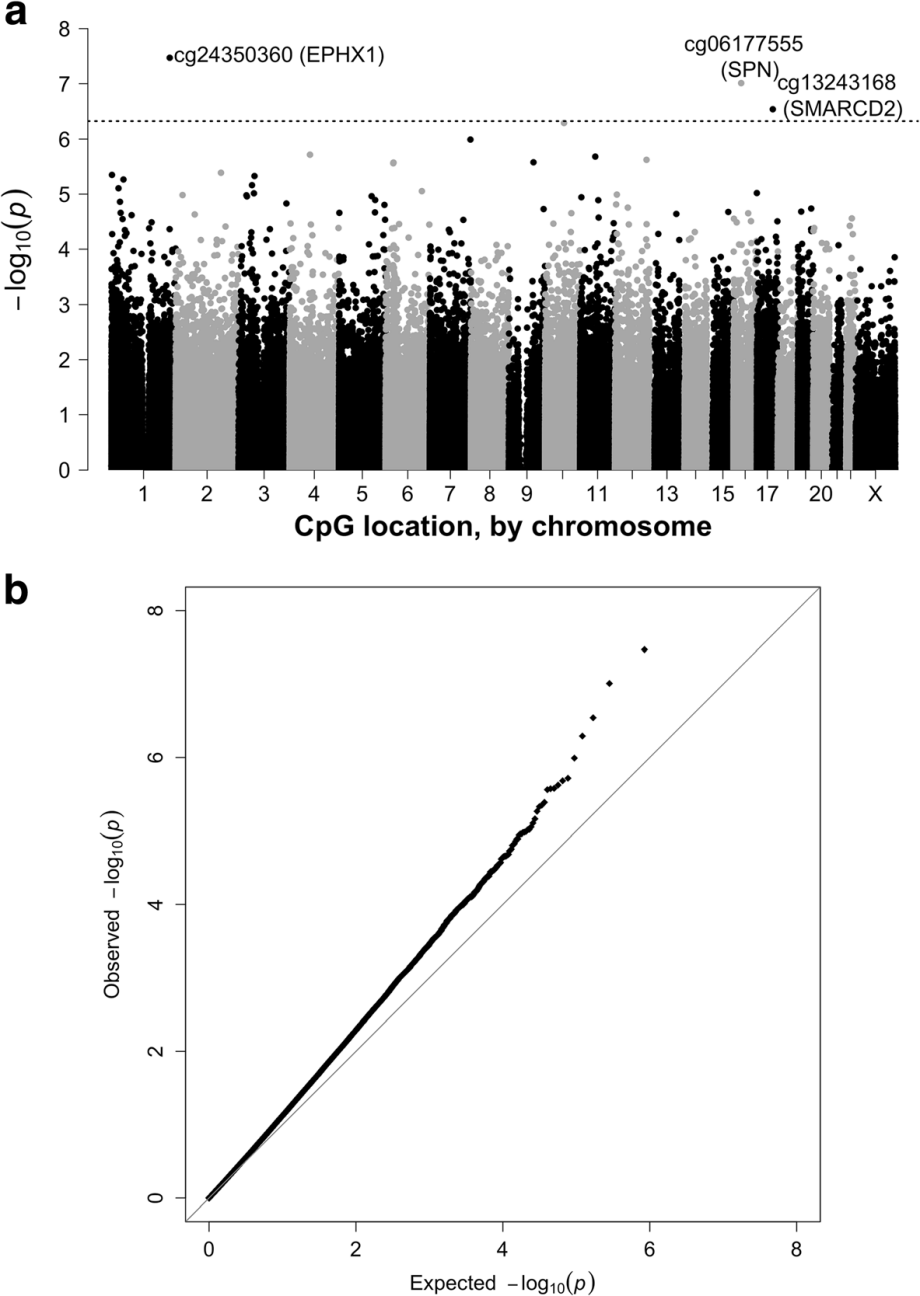
Manhattan plot (**a**) and quantile-quantile plot (**b**) for the associations between serum 25(OH)D levels (modeled continuously) and DNA methylation at 423,500 CpG sites among women in the subcohort (*n* = 1270 non-Hispanic white women). The reference line shows the cut-off for false discovery rate, *q* = 0.05

Within the subcohort, 25(OH)D was associated with methylation levels at three CpGs at *q* < 0.05 (Fig. 2a and Table 3). The CpG with the smallest *p* value was cg24350360 (*EPHX1*; *p* = 3.4 × 10^−8^), followed by cg06177555 (*SPN*; *p* = 9.8 × 10^−8^) and cg1324316 (*SMARCD2*; *p* = 2.9 × 10^−7^). Two other CpGs had *q* < 0.10: cg23761815 (*SLC29A3*; *p* = 5.1 × 10^−7^) and cg10401362 (*DNAJB6*; *p* = 1.0 × 10^−6^). The quantile-quantile plot (Fig. 2b) demonstrates that the observed *p* values systematically deviated from what was expected under the null hypothesis. For all five CpGs with *q* < 0.10, increases in serum 25(OH)D were associated with decreased methylation (Table 3; Additional file 1: Figure S2). All except cg24350360 (*EPHX1*) had ICCs > 0.5.

**Table 3:** CpG sites associated with serum 25(OH)D levels in subcohort (*q* < 0.10)

CpG site	Location (Chr:position)	Location type	Gene	Effect estimate^a^ (95% CI)	*p* value	*q* value
cg24350360	1:225997662^b^	TSS200	*EPHX1*	−0.04 (−0.06 to −0.03)	3.4 × 10^−8^	0.01
cg06177555	16:29678624	3′ UTR	*SPN*	−0.02 (−0.03 to −0.01)	9.8 × 10^−8^	0.02
cg13243168	17:61915833	Body	*SMARCD2*	−0.02 (−0.03 to −0.01)	2.9 × 10^−7^	0.04
cg23761815	10:73083123	Body	*SLC29A3*	−0.02 (−0.03 to −0.01)	5.1 × 10^−7^	0.05
cg10401362	7:157185402	Body	*DNAJB6*	−0.02 (−0.03 to −0.01)	1.0 × 10^−6^	0.09

*CI* confidence interval, *TSS200* within 200 basepairs upstream of the transcription start site, *UTR* untranslated region

^a^Estimated change in methylation (logit(β)) per 10 ng/mL change in serum 25-hydroxyvitamin D (25(OH)D)

^b^Intraclass correlation coefficient < 0.5

**Fig. 3:**
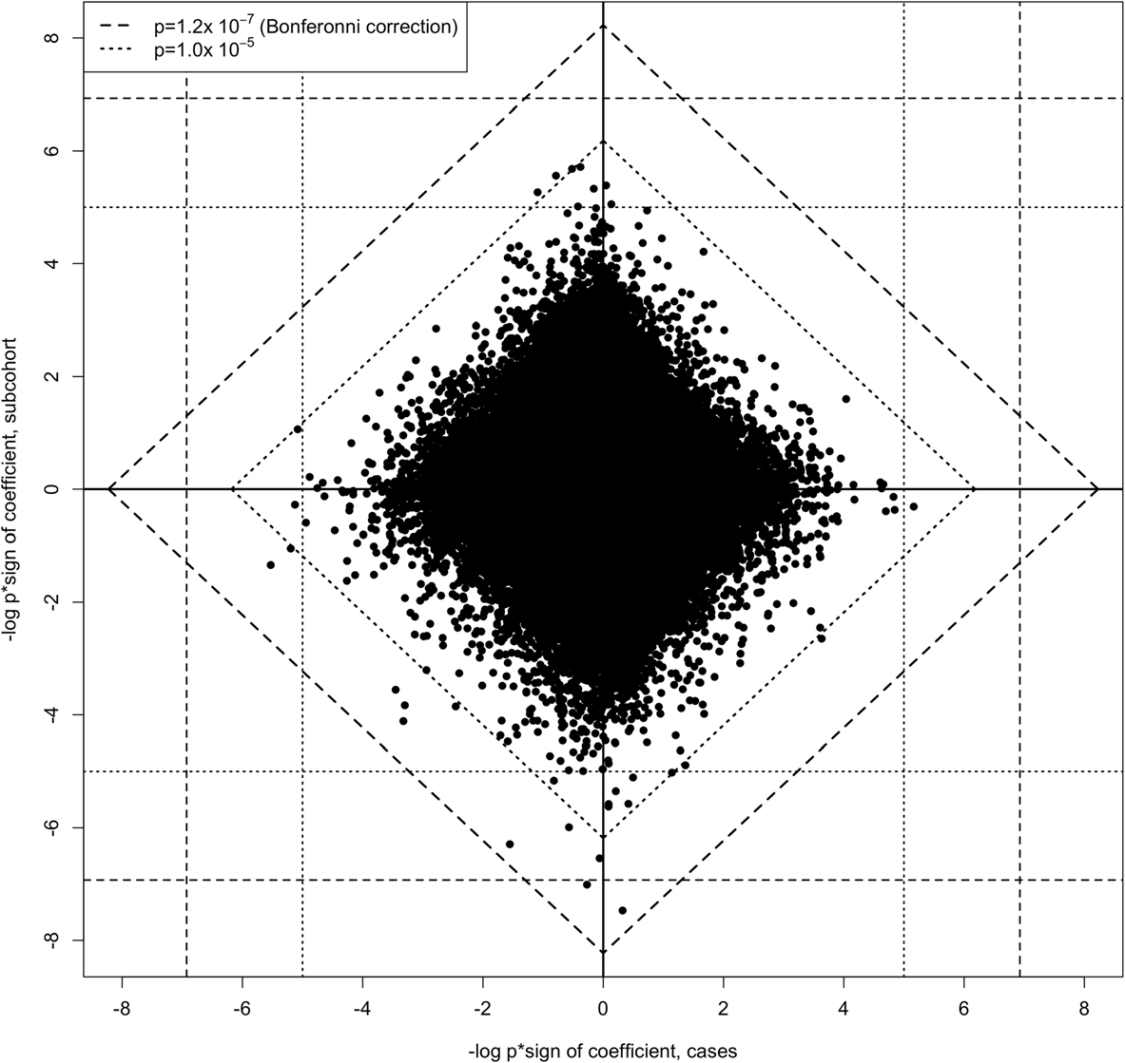
Diamond plot comparing –log_10_ *p* value × sign of coefficient for the estimated association between 25(OH)D and logit(methylation): subcohort (*n* = 1270, including 46 breast cancer cases) versus other breast cancer cases (*n* = 1024). The broken lines show critical values for single (vertical and horizontal grid lines) and Fisher’s combined (diagonal lines) *p* values, based on χ^2^ tests with 2 (for single) and 4 (for combined) degrees of freedom

No CpGs were associated with 25(OH)D in case-only analyses (Additional file 1: Figure S3). When we compared the results of 25(OH)D-methylation association tests for the subcohort versus breast cancer cases (Fig. 3), no CpGs had a combined *p* < 1.2 × 10^−7^, the Bonferroni-corrected cut-point for significance. Sixteen CpGs with combined *p* values < 1.0 × 10^−5^ were deemed worthy of further investigation; all but two of which had ICCs > 0.5 (Table 4). Of the sixteen, nine had RHR *p* values < 0.05. Three of the latter nine were associated with 25(OH)D at *q* < 0.10 in the initial EWAS: cg13243168 (*SMARCD2*), cg23761815 (*SLC29A3*), and cg24350360 (*EPHX1*). Most of the CpGs with small Fisher combined *p* values were inversely associated with 25(OH)D in both cases and the subcohort.

**Table 4:** Ratio of hazard ratios and 95% confidence intervals for the interaction between 25(OH)D and methylation on the risk of breast cancer within 5 years of enrollment (1024 cases, 1270 from subcohort, including 46 additional cases^a^); CpGs with Fisher combined *p* values < 1 × 10^−5^ for subcohort combined with case comparison

Rank	CpG	Gene/ Location	25(OH)D-methylation association, subcohort		25(OH)D-methylation association, cases		Fisher combined *p* value	HR (95% CI) for methylation-breast cancer association	RHRs (95% CI)^c^	Interaction *p* value
			Cofficient^b^	*p* value	Cofficient^b^	*p* value				
1	cg08092930	PPFIA1	−0.03	1.3 × 10^−5^	0.02	0.04	8.5 × 10^−6^*	1.01 (0.98–1.05)	1.15 (1.06–1.24)	6.3 × 10^−4^
2	cg23761815	SLC29A3	−0.02	5.1 × 10^−7^	− 0.01	0.03	2.7 × 10^−7^	1.13 (1.07–1.18)	1.22 (1.08–1.38)	0.002
3	cg13243168	SMARCD2	−0.02	2.9 × 10^−7^	− 7 × 10^−4^	0.87	4.0 × 10^−6^	1.05 (0.98–0.91)	1.29 (1.09–1.51)	0.002
4	cg15544721	PPP1R9A	−0.01	0.09	−0.03	6.4 × 10^−6^	8.8 × 10^−6^	0.98 (0.94–1.03)	0.87 (0.79–0.96)	0.008
5	cg11568290	5p15.1	−0.02	1.4 × 10^−4^	−0.01	0.004	7.7 × 10^−6^	1.15 (1.08–1.22)	1.23 (1.05–1.44)	0.009
6	cg19420720	P4HB	−0.01	0.002	0.02	2.3 × 10^−4^	8.1 × 10^−6^*	1.01 (0.94–1.08)	1.27 (1.06–1.52)	0.01
7	cg23839180	FAM49A	−0.02	0.05	−0.05	3.0 × 10^−6^	2.3 × 10^−6^	0.97 (0.94–1.03)	0.92 (0.86–0.98)	0.01
8	cg15320474^d^	UBD	0.02	2.8 × 10^−6^	− 0.01	0.16	7.0 × 10^−6^*	0.98 (0.83–1.04)	0.87 (0.77–0.99)	0.03
9	cg24350360^d^	EPHX1	−0.04	3.4 × 10^−8^	0.01	0.48	3.1 × 10^−7^*	1.02 (0.99–1.05)	1.08 (1.01–1.16)	0.04
10	cg22488164	PLBD1	−0.03	1.5 × 10^−4^	−0.03	5.0 × 10^−4^	1.3 × 10^−6^	1.06 (1.02–1.10)	0.93 (0.85–1.01)	0.07
11	cg10401362	DNAJB6	−0.02	1.0 × 10^−6^	−0.01	0.27	4.4 × 10^−6^	1.02 (0.97–1.07)	1.10 (0.98–1.24)	0.10
12	cg06177555	SPN	−0.02	9.8 × 10^−8^	−0.003	0.54	9.3 × 10^−7^	0.97 (0.91–1.03)	1.12 (0.98–1.28)	0.10
13	cg11277126	TRPC4AP	−0.02	7.7 × 10^−5^	−0.02	4.8 × 10^−4^	6.7 × 10^−7^	1.04 (0.98–1.11)	1.07 (0.92–1.24)	0.39
14	cg21527411	GLYAT	0.02	2.1 × 10^−6^	−0.01	0.30	9.6 × 10^−6^*	1.02 (0.97–1.07)	0.95 (0.83–1.08)	0.40
15	cg09914444	DMBX1	0.02	5.4 × 10^−6^	−0.01	0.08	6.9 × 10^−6^*	1.05 (1.01–1.10)	0.96 (0.87–1.06)	0.44
16	cg23999318	HIPK2	−0.02	2.8 × 10^−4^	−0.02	3.5 × 10^−4^	1.7 × 10^−6^	1.00 (0.94–1.05)	1.00 (0.88–1.14)	1.00

*CI* confidence interval, *HR* hazard ratio, *RHR* ratio of hazard ratio

^a^After excluding those with missing covariate information

^b^Estimated change in methylation (logit(β)) per 10 ng/mL change in serum 25-hydroxyvitamin D (25(OH)D)

^c^Change in the methylation-breast cancer association for being in the 4th quartile of 25(OH)D (> 38.0 ng/mL) versus the first three (≤ 38.0 ng/mL)—a value > 1.00 indicates that the estimated HR for the methylation-breast cancer association is higher among those with higher 25(OH)D levels; similarly, an RHR < 1.00 indicates that the estimated HR for the methylation-breast cancer association is higher among those with lower 25(OH)D levels

^d^Intraclass correlation coefficient < 0.5

*Effect estimate going in opposing direction for subcohort versus cases

## Discussion

Among our a priori candidate loci, we found that methylation levels at CpGs in or near *RXRA*, *NADSYN1/DHCR7*, and *GC* were associated with serum 25(OH)D levels. In our larger EWAS analysis, CpGs in *EPHX1*, *SPN*, and *SMARCD2* had epigenome-wide significant associations with serum 25(OH)D. To our knowledge, we are the first to report a link between these three genes and 25(OH)D and the first to study methylation-25(OH)D interactions in relation to breast cancer risk.

For candidate CpG analyses, the top hit for both the methylation-25(OH)D association analysis and the breast cancer interaction analyses was cg21201924, located in the gene body of *RXRA*. As previously noted, RXRA acts as a transcription factor with 1,25(OH)_2_D and VDR and changes to the gene’s expression and methylation levels could have widespread biological impacts. Changes in expression or methylation of *GC*, *NADSYN1/DHCR7*, and the other candidate genes may have less pervasive biological effects, but our findings support the hypothesis that these vitamin D-related genes and proteins may interact with circulating vitamin D levels to influence breast cancer risk.

Of the CpGs in or near vitamin D-related genes, most of those that were either associated with 25(OH)D or that showed evidence of interacting with 25(OH)D to affect breast cancer risk were located within gene bodies. Higher 25(OH)D levels tended to be associated with higher methylation, but there was no clear pattern linking CpG locations to the direction of the RHR in the interaction analysis.

None of the candidate CpGs from the vitamin D-related genes met the stringent criterion for statistical significance in the EWAS analysis, and thus there was no overlap between the genes identified in our EWAS and those reported previously to be associated with serum vitamin D levels. One of the two hits reported by Zhu et al. [33] (cg04623955 near *DIO3*) was also assessed in our sample, but we found no evidence of an association (*p* = 0.78). Other CpGs identified in their sample also failed to replicate, including cg23492043 (*p* = 0.64), cg00864867 (*p* = 0.15), and cg16826718 (*p* = 0.62). Eight CpGs reported by Florath et al. [34] were also assessed in our sample, but none were significantly associated with 25(OH)D (*p* values 0.09–0.85). These discrepancies could be related to differences in race, sex, or study design, but could also be the result of sampling variation.

As previously noted, vitamin D plays a role in immune response, including regulation of innate and adaptive immunity [9, 10], as well as detoxification [51]. Possible mechanisms for these actions could be through the 1,25(OH)_2_D-VDR-RXRA complex and its effects on gene transcription [12]. Although there is no established link between 25(OH)D or vitamin D metabolism and *SPN*, *SMARCD2*, *SLC29A3*, or *DNAJB6* specifically, the observed associations between these genes and 25(OH)D could be related to VDR or other components of immune function.

*EPHX1* encodes epoxide hydrolase, an enzyme that breaks down epoxides from xenobiotic aromatic compounds (e.g., polycyclic aromatic hydrocarbons, benzene) [52]. Further, *EPHX1* regulation of detoxification via CYP450 enzymes has been shown to modulate the immune response in mice [53]. Although the direct mechanisms linking *EPHX1* and vitamin D are unclear, an in-vivo study showed that 1,25(OH)_2_D_3_ increased the expression of EPHX1 and other phase I and phase II biotransformation enzymes in the intestinal tissue of vitamin D-deficient rats [54]. There is no known association between *EPHX1* and breast cancer risk [55]. We do note that our results should be interpreted with caution as the *EPHX1* CpG site that was strongly associated with 25(OH)D in our sample had a low ICC (< 0.5), meaning that the within-subject variability was larger than the between-subject variability.

*SPN* encodes a sialoglycoprotein expressed on leukocytes and platelets. Cell culture models have demonstrated that vitamin A and D metabolites upregulate SPN expression [56, 57]. *SMARCD2* encodes a critical component of the SWItch/Sucrose Non-Fermentable (SWI/SNF) chromatin remodeling complex, which uses ATP-derived energy to unwrap or restructure chromatin [58]. SMARCD subunits serve as a link between the SWI/SNF core complex and transcription regulators, including nuclear receptors such as VDR and RXR [59–61]. Although we found no prior reports of a direct link between these sites and breast cancer risk, recent studies have demonstrated that genes encoding for the SWI/SNF chromatin-remodeling complex are mutated in approximately 20% of all human tumors [62] and are considered to be critical tumor suppressors [58] and epigenetic regulators of tumorigenesis [63].

The effect measures we estimate for the interaction analysis, RHRs, measure the extent to which the hazard ratio for the 25(OH)D-breast cancer association depends on the epigenetics, as measured by methylation at a particular CpG locus. Because methylation and 25(OH)D were measured in the same blood samples, we cannot address the temporality of the identified associations to determine whether 25(OH)D influences methylation, methylation influences 25(OH)D, or a third factor influences both. Similarly, we can only assess whether the relationship between methylation and 25(OH)D is different for those who later developed breast cancer, suggesting multiplicative interaction, and not whether 25(OH)D or methylation acts as an effect modifier or mediator. Repeated measures of methylation and 25(OH)D would be needed to address temporality. Future studies could also help to determine the most appropriate cut-point for 25(OH)D levels in gene-by-environment interaction studies. We selected 38.0 ng/mL based on our previous results [3] and other findings supporting a threshold effect of similar magnitude [64], but we cannot be sure what levels have the most biological relevance for breast cancer risk.

We limited our sample to non-Hispanic white women to minimize the influence of genetic ancestry. As such, our results may not be fully generalizable. Our sample is also selective in that all participants had a sister diagnosed with breast cancer, and had, on average, approximately twice the risk of developing breast cancer themselves. Our findings are internally valid, but overrepresented risk factors (e.g., germline genetic or early childhood exposures) may inflate the magnitude of effect estimates if they influence the associations evaluated here.

## Conclusions

Serum 25(OH)D concentrations were associated with methylation levels at candidate CpGs in vitamin D-related genes and three genes with links to immune function or regulation of VDR. Methylation levels of some of these CpGs may interact with vitamin D to affect breast cancer risk. These results contribute to our understanding of the relationship between vitamin D and DNA methylation and the impact of vitamin D on breast cancer risk.

## Additional file

Additional file 1: **Table S1.** Characteristics of participants included in the vitamin D and methylation substudy (Sister Study, 2003–2009); only non-Hispanic white women included. **Table S2.** Associations between 25(OH)D and methylation at CpG sites in vitamin D-related genes (*p* > 0.05); Sister Study subcohort (*n* = 1270). **Table S3.** Interaction effects of 25(OH)D and methylation at CpG sites in vitamin D-related genes on the 5-year risk of breast cancer (1024 cases, 1270 from subcohort, including 46 additional cases): ratio of hazard ratios and 95% confidence intervals for CpGs with interaction *p* values > 0.05. **Table S4.** Interacting effects of 25(OH)D and CpG sites in vitamin D-related genes on the 5-year risk of post-menopausal breast cancer (852 cases, 1026 from subcohort, including 41 additional cases): ratio of hazard ratios and 95% confidence intervals. **Table S5.** Ratio of hazard ratios and 95% confidence intervals for the interaction between 25(OH)D and methylation on the 5-year risk of postmenopausal breast cancer (852 cases, 1026 from subcohort, including 41 additional cases); CpGs with Fisher combined *p* values < 1 × 10^−5^ for subcohort versus case comparison. **Figure S1.** Quantile-quantile plot for the association between the epigenetic-by-25(OH)D interaction term and breast cancer risk among postmenopausal women. **Figure S2.** Volcano plot for the associations between serum 25(OH)D levels (modeled continuously) and DNA methylation at 423,500 CpG sites among 1270 non-Hispanic white women randomly selected from the Sister Study cohort (2003–2009). **Figure S3.** Manhattan plot (top) and quantile-quantile plot (bottom) for the association between DNA methylation at 423,500 CpG sites and serum 25(OH)D among non-Hispanic white women with breast cancer (*n* = 1024; excluding those who were selected as part of subcohort). No CpGs were statistically significant at *q* < 0.05. (DOCX 419 kb)

## Abbreviations

1,25(OH)_2_D: 1,25-Dihydroxyvitamin D
25(OH)D: 25-Hydroxyvitamin D
BMI: Body mass index
CI: Confidence interval
EWAS: Epigenome-wide association study
HR: Hazard ratio
ICC: Intraclass correlation coefficient
LC/MS: Liquid chromatography-mass spectrometry
RHR: Ratio of hazard ratios
RXRA: Retinoid X receptor alpha
SNP: Single nucleotide polymorphism
VDR: Vitamin D receptor
