# Supplementary material for: Vitamin D, DNA methylation, and breast cancer
Source: Breast Cancer Res. 2018 Jul 11;20:70. doi: 10.1186/s13058-018-0994-y (PMC6042268; doi:10.1186/s13058-018-0994-y)

**Table S1: Characteristics of participants included in vitamin D and methylation sub-study (Sister Study, 2003-2009); only non-Hispanic white women included**

| **Characteristic** | **Random subcohort^a^**  **(n= 1,277); N (%)** | **Breast cancer cases^a^ (n=1,070); N (%)** |
| --- | --- | --- |
| **Age at blood draw;** mean (std) | 55.7 (9.0) | 58.7 (8.4) |
| **Total 25(OH)D level;** mean (std) | 32.7 (10.2) | 32.3 (9.8) |
|  |  |  |
| **Total 25(OH)D >38 ng/mL** | 343 (27) | 258 (24) |
| **Regular vitamin D supplement use**  **(>4 times/week)** | 682 (54) | 589 (55) |
| **>1 first degree relative with breast cancer** | 348 (27) | 390 (36) |
| **Education level** |  |  |
| High school or less | 215 (17) | 168 (16) |
| Some college | 430 (34) | 344 (32) |
| Bachelor’s degree | 329 (26) | 274 (26) |
| Graduate degree | 303 (24) | 284 (27) |
| **Post-menopausal** | 864 (68) | 819 (77) |
| **Current body mass index (BMI)** | | |
| <25.0 kg/m^2^ | 511 (40) | 409 (38) |
| 25-29.9 kg/m^2^ | 412 (32) | 343 (32) |
| >30 kg/m^2^ | 352 (28) | 318 (30) |
| **Current hormone therapy use** |  |  |
| None | 1,141 (90) | 916 (86) |
| Estrogen plus progestin | 48 (4) | 62 (6) |
| Unopposed estrogen | 84 (7) | 88 (8) |
| **Current birth control use** | 52 (4) | 37 (3) |
| **Physical Activity (in last year)** | | |
| 0 – 1 hours/week | 420 (33) | 344 (32) |
| 1.1 – 3 hours/week | 398 (31) | 334 (31) |
| >3 hours/week | 459 (36) | 392 (36) |
| **Alcohol consumption in last year** |  |  |
| Never/former drinker | 203 (16) | 175 (16) |
| Current drinker, <1 drink/day | 880 (69) | 725 (68) |
| Current drinker, >1 drink/day | 189 (15) | 170 (16) |
| **Current smoker** | 98 (8) | 73 (7) |
| **Parity** |  |  |
| 0 births | 236 (19) | 204 (19) |
| 1 birth | 170 (13) | 150 (14) |
| 2 births | 475 (37) | 392 (37) |
| >3 births | 395 (31) | 324 (30) |
| **History of osteoporosis** | 328 (26) | 258 (24) |

^a^Includes 46 women who were randomly selected into the subcohort and developed breast cancer within 5 years

Missing values: Regular vitamin D supplement use (23 in subcohort, 7 cases), Menopausal status (1 in subcohort), Current BMI (2 in subcohort), Current hormone therapy use (4 in subcohort, 4 cases), Current birth control use (2 in subcohort, 2 cases), Alcohol (5 in subcohort), Parity (1 in subcohort), Osteoporosis (1 in subcohort)

**Table S2 (continuation of Table 1). Associations between 25(OH)D and methylation at CpG sites in vitamin D-related genes (p>0.05); Sister Study subcohort (n=1,270)**

| **Rank** | **CpG** | **Gene** | **Chromosome: Position** | **Mean methylation level (std)** | **Association with 25(OH)D^a^** | |
| --- | --- | --- | --- | --- | --- | --- |
|  |  |  |  |  | β | p-value |
| 24 | cg08581512^b^ | CYP2R1 | 11: 14913297 | 0.01 (0.002) | 0.008 | 0.06 |
| 25 | cg10592901 | VDR | 12: 48245219 | 0.52 (0.083) | 0.013 | 0.07 |
| 26 | cg13786567 | RXRA | 9: 137258920 | 0.84 (0.018) | -0.006 | 0.07 |
| 27 | cg03137447^b^ | VDR | 12: 48299289 | 0.01 (0.001) | -0.010 | 0.07 |
| 28 | cg27537561^b^ | VDR | 12: 48298656 | 0.01 (0.001) | 0.006 | 0.08 |
| 29 | cg19089328 | NADSYN1 | 11: 71210210 | 0.85 (0.037) | -0.016 | 0.08 |
| 30 | cg11035813^b^ | DHCR7 | 11: 71159731 | 0.01 (0.002) | -0.011 | 0.09 |
| 31 | cg13487983 | RXRA | 9: 137296319 | 0.26 (0.049) | -0.008 | 0.09 |
| 32 | cg14121282 | RXRA | 9: 137268074 | 0.27 (0.055) | -0.011 | 0.09 |
| 33 | cg03589557^b^ | NADSYN1 | 11: 71164283 | 0.04 (0.008) | 0.010 | 0.10 |
| 34 | cg13005613^b^ | RXRA | 9: 137301117 | 0.88 (0.018) | -0.007 | 0.10 |
| 35 | cg13061648^b^ | RXRA | 9: 137331317 | 0.96 (0.006) | -0.008 | 0.10 |
| 36 | cg00324108^b^ | GC | 4: 72635822 | 0.96 (0.007) | 0.009 | 0.10 |
| 37 | cg08854599^b^ | NADSYN1 | 11: 71202779 | 0.98 (0.003) | 0.006 | 0.11 |
| 38 | cg14311020^b^ | VDR | 12: 48298622 | 0.96 (0.008) | -0.001 | 0.12 |
| 39 | cg14545975 | RXRA | 9: 137297213 | 0.75 (0.036) | -0.007 | 0.12 |
| 40 | cg07617163^b^ | NADSYN1 | 11: 71185649 | 0.98 (0.003) | -0.006 | 0.12 |
| 41 | cg13931640 | RXRA | 9: 137277819 | 0.52 (0.060) | -0.006 | 0.13 |
| 42 | cg02220233 | GC | 4: 72669773 | 0.85 (0.036) | 0.012 | 0.13 |
| 43 | cg13865595^b^ | VDR | 12: 48298924 | 0.01 (0.002) | 0.008 | 0.13 |
| 44 | cg10037049 | VDR | 12: 48276633 | 0.06 (0.029) | -0.015 | 0.13 |
| 45 | cg11724562^b^ | NADSYN1 | 11: 71196642 | 0.98 (0.004) | -0.008 | 0.13 |
| 46 | cg04905829 | CYP27B1 | 12: 58160911 | 0.69 (0.045) | 0.008 | 0.13 |
| 47 | cg24164254^b^ | RXRA | 9: 137329341 | 0.92 (0.009) | -0.006 | 0.14 |
| 48 | cg14854850 | VDR | 12: 48237752 | 0.94 (0.012) | 0.009 | 0.14 |
| 49 | cg18787440 | CYP27B1^b^ | 12: 58156782 | 0.96 (0.008) | -0.008 | 0.15 |
| 50 | cg18413900 | CYP27B1 | 12: 58160989 | 0.72 (0.040) | -0.005 | 0.16 |
| 51 | cg13413384 | RXRA | 9: 137302231 | 0.51 (0.069) | -0.004 | 0.17 |
| 52 | cg26528620^b^ | CYP2R1 | 11: 14913351 | 0.02 (0.002) | 0.005 | 0.17 |
| 53 | cg14051721 | RXRA | 9: 137301309 | 0.33 (0.066) | -0.006 | 0.17 |
| 54 | cg07864189^b^ | NADSYN1 | 11: 71164265 | 0.01 (0.002) | 0.008 | 0.17 |
| 55 | cg02470587^b^ | VDR | 12: 48302191 | 0.95 (0.011) | -0.008 | 0.19 |
| 56 | cg00287413 | CYP24A1 | 20: 52788779 | 0.94 (0.024) | 0.014 | 0.21 |
| 57 | cg09816180^b^ | GC | 4: 72650088 | 0.96 (0.006) | -0.006 | 0.21 |
| 58 | cg13941235 | RXRA | 9: 137270186 | 0.48 (0.084) | -0.011 | 0.22 |
| 59 | cg01065422^b^ | NADSYN1 | 11: 71202368 | 0.95 (0.008) | -0.006 | 0.22 |
| 60 | cg14654324^b^ | RXRA | 9: 137299436 | 0.03 (0.006) | 0.007 | 0.22 |
| 61 | cg06523128^b^ | DHCR7 | 11: 71159708 | 0.00 (0.001) | -0.008 | 0.23 |
| 62 | cg03284291^b^ | NADSYN1 | 11: 71202612 | 0.96 (0.007) | -0.007 | 0.24 |
| 63 | cg12960201^b^ | DHCR7 | 11: 71150200 | 0.98 (0.003) | 0.006 | 0.24 |
| 64 | cg22960616^b^ | NADSYN1 | 11: 71188716 | 0.96 (0.007) | -0.006 | 0.25 |
| 65 | cg01604138^b^ | NADSYN1 | 11: 71202794 | 0.93 (0.010) | -0.005 | 0.25 |
| 66 | cg18956481^b^ | CYP24A1 | 20: 52790139 | 0.05 (0.019) | 0.011 | 0.26 |
| 67 | cg25305530^b^ | CYP24A1 | 20: 52793130 | 0.98 (0.004) | 0.004 | 0.26 |
| 68 | cg07185038^b^ | CYP27B1 | 12: 58165658 | 0.05 (0.009) | 0.005 | 0.26 |
| 69 | cg27048527^b^ | NADSYN1 | 11: 71184503 | 0.95 (0.007) | -0.005 | 0.26 |
| 70 | cg19794395 | CYP27B1 | 12: 58158293 | 0.96 (0.006) | -0.006 | 0.27 |
| 71 | cg02604290^b^ | CYP24A1 | 20: 52790589 | 0.03 (0.010) | -0.010 | 0.27 |
| 72 | cg02087383 | GC | 4: 72651102 | 0.94 (0.012) | 0.006 | 0.28 |
| 73 | cg11980188^b^ | CYP2R1 | 11: 14914012 | 0.01 (0.001) | -0.005 | 0.29 |
| 74 | cg25377865 | GC | 4: 72669944 | 0.87 (0.024) | 0.006 | 0.29 |
| 75 | cg10763288 | DHCR7 | 11: 71158925 | 0.55 (0.038) | 0.004 | 0.29 |
| 76 | cg19227606^b^ | NADSYN1 | 11: 71202329 | 0.97 (0.005) | -0.005 | 0.30 |
| 77 | cg14484045^b^ | RXRA | 9: 137265865 | 0.94 (0.010) | -0.005 | 0.30 |
| 78 | cg05163923^b^ | DHCR7 | 11: 71159392 | 0.01 (0.002) | 0.006 | 0.31 |
| 79 | cg10195011^b^ | VDR | 12: 48298798 | 0.01 (0.001) | 0.004 | 0.32 |
| 80 | cg00753924 | RXRA | 9: 137298813 | 0.48 (0.112) | -0.012 | 0.32 |
| 81 | cg14051662^b^ | RXRA | 9: 137279180 | 0.91 (0.014) | -0.004 | 0.33 |
| 82 | cg04321714 | CYP27B1 | 12: 58159999 | 0.41 (0.077) | 0.008 | 0.35 |
| 83 | cg16171723^b^ | DHCR7 | 11: 71150020 | 0.98 (0.002) | -0.004 | 0.35 |
| 84 | cg02411493^b^ | NADSYN1 | 11: 71175151 | 0.99 (0.003) | -0.005 | 0.36 |
| 85 | cg14462321^b^ | RXRA | 9: 137232372 | 0.92 (0.011) | -0.004 | 0.37 |
| 86 | cg20321331^b^ | CYP2R1 | 11: 14913757 | 0.01 (0.002) | -0.007 | 0.38 |
| 87 | cg25588697^b^ | DHCR7 | 11: 71149985 | 0.94 (0.007) | -0.003 | 0.39 |
| 88 | cg07376029 | GC | 4: 72650992 | 0.77 (0.053) | -0.006 | 0.40 |
| 89 | cg19345671^b^ | NADSYN1 | 11: 71164189 | 0.00 (0.000) | 0.003 | 0.41 |
| 90 | cg23101118 | CYP27B1 | 12: 58158888 | 0.05 (0.017) | 0.007 | 0.43 |
| 91 | cg13556224 | VDR | 12: 48299800 | 0.97 (0.010) | 0.007 | 0.43 |
| 92 | cg13936846^b^ | RXRA | 9: 137216064 | 0.90 (0.018) | -0.005 | 0.44 |
| 93 | cg10847948^b^ | NADSYN1 | 11: 71163743 | 0.02 (0.004) | -0.006 | 0.44 |
| 94 | cg13043300^b^ | DHCR7 | 11: 71146211 | 0.98 (0.004) | -0.004 | 0.45 |
| 95 | cg00455178^b^ | CYP2R1 | 11: 14913981 | 0.01 (0.002) | -0.003 | 0.45 |
| 96 | cg02143877^b^ | CYP24A1 | 20: 52790141 | 0.02 (0.004) | -0.002 | 0.46 |
| 97 | cg05454237^b^ | CYP2R1 | 11: 14913849 | 0.01 (0.002) | -0.005 | 0.46 |
| 98 | cg25623977^b^ | NADSYN1 | 11: 71163428 | 0.01 (0.001) | -0.003 | 0.48 |
| 99 | cg08460032^b^ | CYP2R1 | 11: 14909425 | 0.98 (0.003) | 0.003 | 0.49 |
| 100 | cg18313719^b^ | NADSYN1 | 11: 71202112 | 0.96 (0.008) | -0.004 | 0.51 |
| 101 | cg05732940^b^ | CYP27B1 | 12: 58165723 | 0.02 (0.006) | -0.005 | 0.51 |
| 102 | cg17997279 | CYP24A1 | 20: 52791472 | 0.02 (0.006) | -0.006 | 0.52 |
| 103 | cg00545196^b^ | RXRA | 9: 137299685 | 0.91 (0.014) | -0.003 | 0.53 |
| 104 | cg27254468^b^ | NADSYN1 | 11: 71175039 | 0.97 (0.005) | -0.003 | 0.54 |
| 105 | cg12751354 | CYP24A1 | 20: 52770785 | 0.92 (0.019) | 0.004 | 0.54 |
| 106 | cg03018369^b^ | NADSYN1 | 11: 71187445 | 0.99 (0.002) | 0.002 | 0.55 |
| 107 | cg24849555^b^ | CYP24A1 | 20: 52781187 | 0.96 (0.010) | 0.003 | 0.55 |
| 108 | cg17773225^b^ | CYP27B1 | 12: 58165920 | 0.04 (0.013) | 0.006 | 0.55 |
| 109 | cg24341498^b^ | RXRA | 9: 137217390 | 0.03 (0.021) | -0.012 | 0.55 |
| 110 | cg06417365^b^ | NADSYN1 | 11: 71188535 | 0.97 (0.004) | -0.002 | 0.56 |
| 111 | cg24229579^b^ | NADSYN1 | 11: 71189582 | 0.93 (0.011) | -0.003 | 0.57 |
| 112 | cg02399233^b^ | NADSYN1 | 11: 71201915 | 0.98 (0.002) | 0.001 | 0.57 |
| 113 | cg12978433 | CYP24A1 | 20: 52789956 | 0.01 (0.005) | 0.005 | 0.58 |
| 114 | cg17553080^b^ | NADSYN1 | 11: 71164601 | 0.01 (0.002) | -0.004 | 0.59 |
| 115 | cg01476789^b^ | DHCR7 | 11: 71146764 | 0.93 (0.011) | 0.003 | 0.59 |
| 116 | cg01462727 | CYP24A1 | 20: 52786323 | 0.91 (0.036) | -0.007 | 0.59 |
| 117 | cg20006855^b^ | NADSYN1 | 11: 71209518 | 0.98 (0.003) | 0.002 | 0.59 |
| 118 | cg13865488 | RXRA | 9: 137299285 | 0.02 (0.005) | 0.004 | 0.60 |
| 119 | cg11597185^b^ | DHCR7 | 11: 71155081 | 0.97 (0.005) | -0.003 | 0.60 |
| 120 | cg14265220^b^ | RXRA | 9: 137293328 | 0.97 (0.005) | -0.003 | 0.60 |
| 121 | cg27263778^b^ | NADSYN1 | 11: 71201682 | 0.96 (0.006) | 0.003 | 0.61 |
| 122 | cg05072492^b^ | NADSYN1 | 11: 71163907 | 0.01 (0.001) | -0.003 | 0.61 |
| 123 | cg25543401^b^ | NADSYN1 | 11: 71185116 | 0.98 (0.003) | 0.002 | 0.61 |
| 124 | cg13746518 | RXRA | 9: 137332241 | 0.17 (0.047) | 0.005 | 0.64 |
| 125 | cg02972188^b^ | CYP27B1 | 12: 58165945 | 0.03 (0.008) | -0.004 | 0.64 |
| 126 | cg14204281^b^ | RXRA | 9: 137217473 | 0.02 (0.004) | 0.003 | 0.64 |
| 127 | cg12090177^b^ | NADSYN1 | 11: 71202719 | 0.98 (0.003) | 0.002 | 0.65 |
| 128 | cg06169276^b^ | NADSYN1 | 11: 71164262 | 0.01 (0.002) | -0.001 | 0.65 |
| 129 | cg03307911^b^ | CYP24A1 | 20: 52790601 | 0.04 (0.006) | 0.002 | 0.65 |
| 130 | cg05896371^b^ | DHCR7 | 11: 71159218 | 0.01 (0.002) | -0.002 | 0.66 |
| 131 | cg04875697^b^ | RXRA | 9: 137228542 | 0.97 (0.005) | -0.002 | 0.66 |
| 132 | cg23792245^b^ | CYP2R1 | 11: 14913752 | 0.04 (0.008) | 0.002 | 0.66 |
| 133 | cg06326648^b^ | CYP2R1 | 11: 14912680 | 0.01 (0.002) | 0.002 | 0.67 |
| 134 | cg23685712 | CYP24A1 | 20: 52790063 | 0.01 (0.003) | -0.004 | 0.68 |
| 135 | cg23305502^b^ | NADSYN1 | 11: 71164098 | 0.00 (0.000) | 0.001 | 0.70 |
| 136 | cg04946948^b^ | NADSYN1 | 11: 71196653 | 0.96 (0.007) | 0.002 | 0.70 |
| 137 | cg09253762 | CYP27B1 | 12: 58165392 | 0.27 (0.040) | -0.002 | 0.71 |
| 138 | cg14468605^b^ | RXRA | 9: 137255666 | 0.97 (0.003) | 0.001 | 0.71 |
| 139 | cg05240381^b^ | NADSYN1 | 11: 71164349 | 0.03 (0.006) | -0.004 | 0.72 |
| 140 | cg01182309 | CYP27B1 | 12: 58159692 | 0.03 (0.020) | 0.007 | 0.73 |
| 141 | cg14022501 | RXRA | 9: 137335080 | 0.53 (0.049) | -0.002 | 0.73 |
| 142 | cg14344989^b^ | RXRA | 9: 137329237 | 0.01 (0.002) | 0.009 | 0.74 |
| 143 | cg12474705^b^ | NADSYN1 | 11: 71193499 | 0.96 (0.007) | -0.002 | 0.74 |
| 144 | cg21843272 | VDR | 12: 48235729 | 0.97 (0.009) | 0.003 | 0.75 |
| 145 | cg01630362^b^ | NADSYN1 | 11: 71163528 | 0.02 (0.004) | -0.002 | 0.78 |
| 146 | cg25454890^b^ | CYP2R1 | 11: 14913620 | 0.02 (0.006) | 0.002 | 0.78 |
| 147 | cg25219939^b^ | VDR | 12: 48250774 | 0.98 (0.004) | -0.001 | 0.79 |
| 148 | cg07487535^b^ | DHCR7 | 11: 71159905 | 0.02 (0.004) | 0.002 | 0.79 |
| 149 | cg24110768^b^ | CYP27B1 | 12: 58159478 | 0.02 (0.004) | 0.002 | 0.79 |
| 150 | cg23654431^b^ | VDR | 12: 48298967 | 0.02 (0.004) | 0.002 | 0.80 |
| 151 | cg02712555^b^ | CYP24A1 | 20: 52790733 | 0.01 (0.002) | 0.001 | 0.80 |
| 152 | cg05215649^b^ | NADSYN1 | 11: 71196591 | 0.92 (0.010) | -0.001 | 0.80 |
| 153 | cg02547054 | VDR | 12: 48296637 | 0.82 (0.022) | -0.001 | 0.80 |
| 154 | cg13847322 | RXRA | 9: 137272766 | 0.82 (0.034) | -0.001 | 0.81 |
| 155 | cg06146607^b^ | NADSYN1 | 11: 71167505 | 0.88 (0.016) | -0.001 | 0.81 |
| 156 | cg11227966^b^ | CYP2R1 | 11: 14917425 | 0.96 (0.007) | -0.001 | 0.81 |
| 157 | cg08752080^b^ | GC | 4: 72631312 | 0.93 (0.012) | -0.001 | 0.82 |
| 158 | cg06369854^b^ | VDR | 12: 48299313 | 0.01 (0.002) | -0.002 | 0.82 |
| 159 | cg13978325^b^ | RXRA | 9: 137218288 | 0.02 (0.008) | -0.002 | 0.83 |
| 160 | cg21560157^b^ | NADSYN1 | 11: 71212402 | 0.98 (0.002) | 0.001 | 0.83 |
| 161 | cg02622885 ^b^ | CYP2R1 | 11: 14913424 | 0.03 (0.006) | 0.001 | 0.85 |
| 162 | cg05963690^b^ | NADSYN1 | 11: 71163466 | 0.01 (0.001) | 0.001 | 0.86 |
| 163 | cg01385795^b^ | CYP2R1 | 11: 14913124 | 0.02 (0.006) | 0.001 | 0.87 |
| 164 | cg13425135 | RXRA | 9: 137330570 | 0.99 (0.002) | 0.000 | 0.87 |
| 165 | cg07060721 | CYP27B1 | 12: 58158537 | 0.12 (0.033) | -0.001 | 0.88 |
| 166 | cg03544918^b^ | CYP2R1 | 11: 14913944 | 0.01 (0.001) | -0.001 | 0.88 |
| 167 | cg16984335^b^ | CYP27B1 | 12: 58159051 | 0.01 (0.002) | -0.001 | 0.88 |
| 168 | cg20733846^b^ | NADSYN1 | 11: 71209416 | 0.97 (0.003) | 0.000 | 0.89 |
| 169 | cg09800519^b^ | RXRA | 9: 137218286 | 0.02 (0.007) | -0.001 | 0.89 |
| 170 | cg02673418^b^ | CYP24A1 | 20: 52790978 | 0.01 (0.002) | 0.001 | 0.89 |
| 171 | cg02121892^b^ | NADSYN1 | 11: 71174976 | 0.98 (0.004) | 0.001 | 0.90 |
| 172 | cg05220083^b^ | CYP27B1 | 12: 58152793 | 0.95 (0.006) | -0.001 | 0.90 |
| 173 | cg15757022^b^ | NADSYN1 | 11: 71163853 | 0.00 (0.001) | -0.001 | 0.90 |
| 174 | cg11383802^b^ | DHCR7 | 11: 71145904 | 0.98 (0.002) | 0.000 | 0.90 |
| 175 | cg18482822^b^ | DHCR7 | 11: 71146456 | 0.97 (0.005) | 0.001 | 0.91 |
| 176 | cg24722099 | CYP27B1 | 12: 58160843 | 0.36 (0.044) | 0.000 | 0.91 |
| 177 | cg14570632^b^ | RXRA | 9: 137243592 | 0.96 (0.006) | 0.000 | 0.92 |
| 178 | cg21201884^b^ | NADSYN1 | 11: 71183261 | 0.97 (0.006) | 0.000 | 0.93 |
| 179 | cg06883848^b^ | CYP27B1 | 12: 58160421 | 0.03 (0.010) | 0.001 | 0.93 |
| 180 | cg07212292^b^ | NADSYN1 | 11: 71164112 | 0.00 (0.001) | 0.000 | 0.93 |
| 181 | cg16544887^b^ | CYP2R1 | 11: 14913979 | 0.01 (0.002) | 0.000 | 0.93 |
| 182 | cg13689699^b^ | RXRA | 9: 137225311 | 0.97 (0.005) | 0.000 | 0.93 |
| 183 | cg16408394^b^ | RXRA | 9: 137219075 | 0.01 (0.002) | 0.000 | 0.94 |
| 184 | cg13595495^b^ | RXRA | 9: 137279412 | 0.97 (0.005) | 0.000 | 0.95 |
| 185 | cg19008693^b^ | VDR | 12: 48270864 | 0.98 (0.003) | 0.000 | 0.95 |
| 186 | cg04460185^b^ | DHCR7 | 11: 71160219 | 0.01 (0.001) | 0.000 | 0.95 |
| 187 | cg24582168 | CYP24A1 | 20: 52789646 | 0.04 (0.010) | 0.000 | 0.95 |
| 188 | cg16321474^b^ | VDR | 12: 48299386 | 0.01 (0.004) | -0.001 | 0.96 |
| 189 | cg14472716 | RXRA | 9: 137236548 | 0.29 (0.043) | 0.000 | 0.96 |
| 190 | cg16802027^b^ | CYP2R1 | 11: 14913860 | 0.04 (0.010) | 0.000 | 0.97 |
| 191 | cg18210221^b^ | DHCR7 | 11: 71150315 | 0.99 (0.001) | 0.000 | 0.97 |
| 192 | cg09290506^b^ | CYP27B1 | 12: 58165961 | 0.00 (0.000) | 0.000 | 0.97 |
| 193 | cg24826892^b^ | DHCR7 | 11: 71159390 | 0.01 (0.001) | 0.000 | 0.97 |
| 194 | cg00630245^b^ | DHCR7 | 11: 71146831 | 0.91 (0.013) | 0.000 | 0.98 |
| 195 | cg01892327^b^ | DHCR7 | 11: 71159316 | 0.01 (0.001) | 0.000 | 0.98 |
| 196 | cg02319187^b^ | RXRA | 9: 137229907 | 0.96 (0.008) | 0.000 | 0.99 |
| 197 | cg13677043 | RXRA | 9: 137253049 | 0.94 (0.013) | 0.000 | 0.99 |
| 198 | cg13855454 | VDR | 12: 48232358 | 0.91 (0.015) | 0.000 | 1.00 |

^a^Estimated change in methylation (logit(β)) per 10 ng/mL change in serum 25(OH)D

^b^Intraclass correlation coefficient for probe <0.5

**Table S3 (continuation of Table 2). Interaction effects of 25(OH)D and methylation at CpG sites in vitamin D-related genes on the 5-year risk of breast cancer (1024 cases, 1270 from subcohort, including 46 additional cases^a^): Ratio of hazard ratios and 95% confidence intervals for CpGs with interaction p-values >0.05)**

| **Rank** | **CpG site** | **Gene** | **HR (95% CI) for methylation-breast cancer association** | **HR (95% CI) for methylation-breast cancer association, if 25(OH)D <38.0 ng/mL** | **HR (95% CI) for methylation-breast cancer association, if 25OH)D >38.0 ng/mL** | **Ratio of Hazard Ratios**  **(95% CI)^b^** | **Inter-action p-value** |
| --- | --- | --- | --- | --- | --- | --- | --- |
| 19 | cg23101118 | CYP27B1 | 1.02 (0.99, 1.05) | 1.00 (0.97, 1.03) | 1.07 (1.01, 1.13) | 1.07 (1.00, 1.14) | 0.05 |
| 20 | cg19345671^c^ | NADSYN1 | 1.02 (0.94, 1.10) | 1.06 (0.97, 1.16) | 0.88 (0.75, 1.04) | 0.84 (0.69, 1.01) | 0.06 |
| 21 | cg00753924 | RXRA | 1.00 (0.99, 1.02) | 1.00 (0.98, 1.02) | 1.04 (1.00, 1.08) | 1.04 (1.00, 1.09) | 0.06 |
| 22 | cg14121282 | RXRA | 1.04 (0.99, 1.08) | 1.01 (0.96, 1.07) | 1.12 (1.02, 1.22) | 1.10 (0.99, 1.22) | 0.06 |
| 23 | cg01630362^c^ | NADSYN1 | 0.96 (0.92, 1.00) | 0.98 (0.93, 1.04) | 0.89 (0.81, 0.97) | 0.91 (0.81, 1.01) | 0.06 |
| 24 | cg17559402^c^ | NADSYN1 | 0.96 (0.92, 1.00) | 0.94 (0.89, 0.98) | 1.02 (0.94, 1.11) | 1.09 (0.99, 1.20) | 0.07 |
| 25 | cg05785753 | NADSYN1 | 0.97 (0.93, 1.00) | 0.94 (0.90, 0.99) | 1.02 (0.95, 1.10) | 1.08 (0.99, 1.17) | 0.07 |
| 26 | cg02470587^c^ | VDR | 1.03 (0.99, 1.07) | 1.01 (0.97, 1.06) | 1.10 (1.01, 1.20) | 1.09 (0.99, 1.20) | 0.08 |
| 27 | cg13413384 | RXRA | 1.01 (0.93, 1.10) | 0.97 (0.88, 1.07) | 1.14 (0.97, 1.34) | 1.17 (0.97, 1.42) | 0.10 |
| 28 | cg07376029 | GC | 1.03 (1.00, 1.07) | 1.02 (0.97, 1.06) | 1.09 (1.01, 1.17) | 1.07 (0.99, 1.16) | 0.10 |
| 29 | cg07487535^c^ | DHCR7 | 1.04 (1.00, 1.08) | 1.03 (0.99, 1.08) | 1.11 (1.02, 1.20) | 1.08 (0.98, 1.18) | 0.11 |
| 30 | cg13043300^c^ | DHCR7 | 1.01 (0.97, 1.06) | 0.99 (0.94, 1.04) | 1.08 (0.98, 1.18) | 1.09 (0.98, 1.21) | 0.11 |
| 31 | cg27263778^c^ | NADSYN1 | 0.96 (0.91, 1.01) | 0.98 (0.93, 1.04) | 0.89 (0.79, 0.99) | 0.90 (0.80, 1.03) | 0.12 |
| 32 | cg09997530 | GC | 1.04 (0.99, 1.09) | 1.06 (1.01, 1.12) | 0.97 (0.88, 1.07) | 0.91 (0.82, 1.02) | 0.12 |
| 33 | cg23305502^c^ | NADSYN1 | 1.03 (0.94, 1.12) | 1.07 (0.97, 1.18) | 0.90 (0.74, 1.09) | 0.84 (0.68, 1.05) | 0.12 |
| 34 | cg13746518 | RXRA | 1.02 (1.00, 1.05) | 1.01 (0.98, 1.04) | 1.07 (1.01, 1.14) | 1.05 (0.99, 1.13) | 0.13 |
| 35 | cg19227606^c^ | NADSYN1 | 0.93 (0.88, 0.97) | 0.90 (0.85, 0.96) | 0.99 (0.89, 1.11) | 1.10 (0.97, 1.25) | 0.13 |
| 36 | cg05220083^c^ | CYP27B1 | 1.03 (0.96, 1.10) | 1.00 (0.92, 1.09) | 1.13 (0.99, 1.29) | 1.13 (0.96, 1.32) | 0.13 |
| 37 | cg16151558^c^ | DHCR7 | 1.04 (1.00, 1.08) | 1.06 (1.01, 1.11) | 0.98 (0.90, 1.07) | 0.93 (0.84, 1.02) | 0.14 |
| 38 | cg09290506^c^ | CYP27B1 | 1.05 (0.97, 1.14) | 1.02 (0.93, 1.12) | 1.17 (0.99, 1.38) | 1.15 (0.95, 1.39) | 0.16 |
| 39 | cg03284291^c^ | NADSYN1 | 0.99 (0.95, 1.04) | 0.98 (0.92, 1.03) | 1.06 (0.96, 1.17) | 1.08 (0.97, 1.21) | 0.16 |
| 40 | cg03146219 | NADSYN1 | 0.97 (0.94, 1.00) | 0.96 (0.92, 0.99) | 1.01 (0.95, 1.07) | 1.05 (0.98, 1.13) | 0.16 |
| 41 | cg14468605^c^ | RXRA | 1.04 (0.97, 1.13) | 1.07 (0.98, 1.18) | 0.95 (0.81, 1.10) | 0.88 (0.74, 1.05) | 0.16 |
| 42 | cg04460185^c^ | DHCR7 | 0.99 (0.95, 1.04) | 0.97 (0.92, 1.02) | 1.04 (0.96, 1.14) | 1.07 (0.97, 1.19) | 0.16 |
| 43 | cg04905829 | CYP27B1 | 1.06 (1.01, 1.11) | 1.08 (1.02, 1.13) | 0.99 (0.88, 1.10) | 0.92 (0.81, 1.04) | 0.16 |
| 44 | cg01065422^c^ | NADSYN1 | 0.96 (0.91, 1.01) | 0.94 (0.89, 1.00) | 1.02 (0.93, 1.12) | 1.08 (0.97, 1.21) | 0.16 |
| 45 | cg02059519 | RXRA | 1.02 (0.97, 1.08) | 1.00 (0.93, 1.06) | 1.10 (0.97, 1.24) | 1.10 (0.96, 1.26) | 0.17 |
| 46 | cg15757022^c^ | NADSYN1 | 1.02 (0.97, 1.08) | 1.03 (0.97, 1.10) | 0.93 (0.81, 1.07) | 0.90 (0.77, 1.05) | 0.17 |
| 47 | cg14311020^c^ | VDR | 1.03 (0.96, 1.10) | 1.01 (0.93, 1.10) | 1.13 (0.99, 1.30) | 1.12 (0.95, 1.32) | 0.17 |
| 48 | cg04321714 | CYP27B1 | 1.02 (0.99, 1.04) | 1.02 (0.99, 1.05) | 0.97 (0.91, 1.04) | 0.95 (0.89, 1.02) | 0.18 |
| 49 | cg16171723^c^ | DHCR7 | 0.98 (0.93, 1.04) | 0.97 (0.90, 1.04) | 1.07 (0.94, 1.21) | 1.10 (0.96, 1.27) | 0.18 |
| 50 | cg04774822 | NADSYN1 | 1.03 (0.97, 1.10) | 1.02 (0.95, 1.09) | 1.12 (0.99, 1.28) | 1.11 (0.95, 1.28) | 0.18 |
| 51 | cg14204281^c^ | RXRA | 1.03 (1.00, 1.07) | 1.05 (1.01, 1.10) | 0.99 (0.91, 1.07) | 0.94 (0.86, 1.03) | 0.19 |
| 52 | cg06146607^c^ | NADSYN1 | 1.03 (0.98, 1.09) | 1.00 (0.94, 1.07) | 1.09 (0.97, 1.22) | 1.09 (0.96, 1.24) | 0.21 |
| 53 | cg11597185^c^ | DHCR7 | 0.98 (0.94, 1.02) | 1.00 (0.94, 1.05) | 0.93 (0.85, 1.02) | 0.94 (0.84, 1.04) | 0.21 |
| 54 | cg08460032^c^ | CYP2R1 | 0.93 (0.88, 0.98) | 0.91 (0.85, 0.96) | 0.99 (0.88, 1.11) | 1.09 (0.95, 1.24) | 0.21 |
| 55 | cg04329455^c^ | RXRA | 1.01 (0.96, 1.05) | 1.02 (0.97, 1.08) | 0.95 (0.86, 1.05) | 0.93 (0.83, 1.04) | 0.21 |
| 56 | cg24849555^c^ | CYP24A1 | 1.00 (0.96, 1.04) | 0.98 (0.94, 1.03) | 1.05 (0.96, 1.15) | 1.07 (0.96, 1.19) | 0.23 |
| 57 | cg24722099 | CYP27B1 | 1.01 (0.95, 1.06) | 1.02 (0.96, 1.08) | 0.93 (0.81, 1.06) | 0.91 (0.79, 1.06) | 0.23 |
| 58 | cg03137447^c^ | VDR | 0.99 (0.95, 1.03) | 0.97 (0.93, 1.03) | 1.04 (0.95, 1.13) | 1.07 (0.96, 1.18) | 0.23 |
| 59 | cg07617163^c^ | NADSYN1 | 1.01 (0.95, 1.08) | 0.99 (0.93, 1.06) | 1.09 (0.95, 1.24) | 1.10 (0.94, 1.27) | 0.23 |
| 60 | cg00630245^c^ | DHCR7 | 0.94 (0.89, 0.99) | 0.96 (0.90, 1.02) | 0.89 (0.79, 1.00) | 0.92 (0.81, 1.06) | 0.25 |
| 61 | cg02712555^c^ | CYP24A1 | 0.99 (0.94, 1.03) | 0.98 (0.93, 1.03) | 1.04 (0.95, 1.15) | 1.07 (0.95, 1.19) | 0.26 |
| 62 | cg01892327^c^ | DHCR7 | 1.01 (0.97, 1.06) | 1.03 (0.97, 1.08) | 0.96 (0.88, 1.06) | 0.94 (0.85, 1.05) | 0.26 |
| 63 | cg14051662^c^ | RXRA | 1.01 (0.96, 1.07) | 1.00 (0.94, 1.06) | 1.07 (0.96, 1.20) | 1.07 (0.95, 1.22) | 0.27 |
| 64 | cg08854599^c^ | NADSYN1 | 1.00 (0.93, 1.06) | 1.01 (0.93, 1.09) | 0.92 (0.80, 1.06) | 0.91 (0.78, 1.07) | 0.27 |
| 65 | cg12751354 | CYP24A1 | 0.90 (0.86, 0.93) | 0.90 (0.86, 0.95) | 0.86 (0.79, 0.93) | 0.95 (0.87, 1.04) | 0.27 |
| 66 | cg13061648^c^ | RXRA | 0.99 (0.95, 1.05) | 1.01 (0.95, 1.07) | 0.95 (0.85, 1.06) | 0.93 (0.82, 1.06) | 0.28 |
| 67 | cg14472716 | RXRA | 1.02 (0.96, 1.07) | 1.03 (0.96, 1.10) | 0.96 (0.86, 1.07) | 0.93 (0.82, 1.06) | 0.28 |
| 68 | cg16802027^c^ | CYP2R1 | 0.99 (0.95, 1.03) | 1.00 (0.95, 1.04) | 0.94 (0.86, 1.03) | 0.95 (0.86, 1.05) | 0.29 |
| 69 | cg06369854^c^ | VDR | 1.01 (0.98, 1.05) | 1.01 (0.97, 1.05) | 1.05 (0.98, 1.14) | 1.05 (0.96, 1.14) | 0.30 |
| 70 | cg13978325^c^ | RXRA | 1.06 (1.03, 1.09) | 1.05 (1.01, 1.08) | 1.09 (1.02, 1.15) | 1.04 (0.97, 1.11) | 0.30 |
| 71 | cg22960616^c^ | NADSYN1 | 1.00 (0.95, 1.05) | 0.98 (0.93, 1.04) | 1.04 (0.95, 1.15) | 1.06 (0.95, 1.19) | 0.31 |
| 72 | cg08581512^c^ | CYP2R1 | 0.95 (0.89, 1.01) | 0.96 (0.90, 1.04) | 0.89 (0.78, 1.02) | 0.92 (0.79, 1.08) | 0.31 |
| 73 | cg13936846^c^ | RXRA | 1.00 (0.96, 1.04) | 0.99 (0.94, 1.04) | 1.04 (0.95, 1.13) | 1.05 (0.95, 1.17) | 0.31 |
| 74 | cg01182309 | CYP27B1 | 0.99 (0.98, 1.00) | 1.00 (0.98, 1.01) | 0.98 (0.95, 1.01) | 0.98 (0.95, 1.02) | 0.32 |
| 75 | cg11227966^c^ | CYP2R1 | 0.95 (0.89, 1.00) | 0.95 (0.89, 1.02) | 0.90 (0.80, 1.01) | 0.94 (0.82, 1.07) | 0.35 |
| 76 | cg16321474^c^ | VDR | 1.01 (0.98, 1.04) | 1.01 (0.98, 1.05) | 0.98 (0.93, 1.04) | 0.97 (0.91, 1.03) | 0.35 |
| 77 | cg10037049 | VDR | 0.98 (0.96, 1.01) | 0.99 (0.96, 1.01) | 0.96 (0.91, 1.01) | 0.97 (0.92, 1.03) | 0.36 |
| 78 | cg07793224^c^ | NADSYN1 | 1.00 (0.96, 1.04) | 1.01 (0.96, 1.06) | 0.97 (0.89, 1.05) | 0.96 (0.86, 1.05) | 0.36 |
| 79 | cg18413900 | CYP27B1 | 1.05 (0.98, 1.12) | 1.03 (0.96, 1.11) | 1.11 (0.96, 1.27) | 1.07 (0.92, 1.26) | 0.37 |
| 80 | cg13556224 | VDR | 1.03 (1.00, 1.06) | 1.04 (1.00, 1.07) | 1.00 (0.94, 1.07) | 0.97 (0.90, 1.04) | 0.37 |
| 81 | cg19089328 | NADSYN1 | 1.01 (0.98, 1.04) | 1.00 (0.97, 1.03) | 1.03 (0.97, 1.09) | 1.03 (0.96, 1.10) | 0.37 |
| 82 | cg16544887^c^ | CYP2R1 | 1.00 (0.96, 1.05) | 1.01 (0.96, 1.07) | 0.96 (0.87, 1.06) | 0.95 (0.85, 1.06) | 0.38 |
| 83 | cg18787440^c^ | CYP27B1 | 0.94 (0.90, 0.99) | 0.94 (0.89, 1.00) | 0.89 (0.81, 0.99) | 0.95 (0.84, 1.07) | 0.39 |
| 84 | cg16408394^c^ | RXRA | 1.00 (0.97, 1.04) | 1.01 (0.96, 1.06) | 0.97 (0.90, 1.05) | 0.96 (0.88, 1.05) | 0.39 |
| 85 | cg13687497 | RXRA | 1.02 (0.95, 1.09) | 1.00 (0.92, 1.08) | 1.07 (0.94, 1.22) | 1.07 (0.92, 1.24) | 0.41 |
| 86 | cg02399233^c^ | NADSYN1 | 1.00 (0.91, 1.10) | 1.02 (0.92, 1.14) | 0.93 (0.76, 1.13) | 0.91 (0.72, 1.14) | 0.41 |
| 87 | cg02121892^c^ | NADSYN1 | 0.96 (0.91, 1.00) | 0.96 (0.91, 1.01) | 0.91 (0.82, 1.02) | 0.95 (0.85, 1.07) | 0.41 |
| 88 | cg07864189^c^ | NADSYN1 | 1.04 (1.00, 1.08) | 1.05 (1.00, 1.10) | 1.01 (0.92, 1.10) | 0.96 (0.87, 1.06) | 0.41 |
| 89 | cg03589557^c^ | NADSYN1 | 0.98 (0.94, 1.03) | 0.99 (0.94, 1.04) | 0.94 (0.86, 1.04) | 0.96 (0.86, 1.06) | 0.41 |
| 90 | cg17553080^c^ | NADSYN1 | 0.97 (0.94, 1.01) | 0.96 (0.92, 1.01) | 1.00 (0.92, 1.08) | 1.04 (0.95, 1.14) | 0.42 |
| 91 | cg18210221^c^ | DHCR7 | 1.00 (0.90, 1.10) | 0.99 (0.88, 1.12) | 1.09 (0.90, 1.33) | 1.10 (0.87, 1.39) | 0.42 |
| 92 | cg24110768^c^ | CYP27B1 | 0.98 (0.94, 1.01) | 0.97 (0.92, 1.01) | 1.00 (0.93, 1.07) | 1.03 (0.95, 1.13) | 0.43 |
| 93 | cg13865488 | RXRA | 1.02 (0.99, 1.06) | 1.03 (0.99, 1.08) | 0.99 (0.91, 1.08) | 0.96 (0.88, 1.06) | 0.43 |
| 94 | cg01385795^c^ | CYP2R1 | 0.99 (0.96, 1.01) | 0.99 (0.96, 1.02) | 0.96 (0.91, 1.03) | 0.97 (0.91, 1.04) | 0.43 |
| 95 | cg03307911^c^ | CYP24A1 | 0.98 (0.94, 1.04) | 0.97 (0.92, 1.03) | 1.02 (0.92, 1.15) | 1.05 (0.93, 1.19) | 0.43 |
| 96 | cg26528620^c^ | CYP2R1 | 0.98 (0.92, 1.03) | 0.99 (0.93, 1.06) | 0.94 (0.84, 1.05) | 0.95 (0.83, 1.08) | 0.44 |
| 97 | cg13595495^c^ | RXRA | 0.95 (0.91, 1.00) | 0.94 (0.89, 0.99) | 0.98 (0.89, 1.09) | 1.05 (0.93, 1.18) | 0.44 |
| 98 | cg14344989^c^ | RXRA | 0.98 (0.94, 1.03) | 1.00 (0.95, 1.05) | 0.96 (0.87, 1.05) | 0.96 (0.87, 1.07) | 0.44 |
| 99 | cg24341498^c^ | RXRA | 1.00 (0.99, 1.01) | 1.00 (0.99, 1.01) | 1.01 (0.98, 1.04) | 1.01 (0.98, 1.05) | 0.45 |
| 100 | cg13865595^c^ | VDR | 0.99 (0.94, 1.03) | 1.00 (0.95, 1.05) | 0.96 (0.87, 1.05) | 0.96 (0.86, 1.07) | 0.45 |
| 101 | cg18313719^c^ | NADSYN1 | 0.99 (0.95, 1.03) | 0.98 (0.93, 1.03) | 1.02 (0.92, 1.13) | 1.04 (0.93, 1.17) | 0.45 |
| 102 | cg02622885^c^ | CYP2R1 | 0.97 (0.93, 1.01) | 0.97 (0.93, 1.02) | 0.94 (0.86, 1.03) | 0.96 (0.87, 1.07) | 0.46 |
| 103 | cg05963690^c^ | NADSYN1 | 0.98 (0.94, 1.03) | 0.99 (0.94, 1.04) | 0.95 (0.85, 1.05) | 0.96 (0.85, 1.08) | 0.46 |
| 104 | cg13677043 | RXRA | 1.05 (1.00, 1.12) | 1.04 (0.98, 1.12) | 1.09 (0.98, 1.22) | 1.05 (0.92, 1.19) | 0.48 |
| 105 | cg10763288 | DHCR7 | 1.04 (0.98, 1.12) | 1.03 (0.95, 1.11) | 1.09 (0.94, 1.26) | 1.06 (0.90, 1.25) | 0.49 |
| 106 | cg24164254^c^ | RXRA | 1.02 (0.95, 1.09) | 1.00 (0.92, 1.08) | 1.06 (0.91, 1.24) | 1.06 (0.89, 1.27) | 0.49 |
| 107 | cg07099121 ^c^ | DHCR7 | 0.94 (0.89, 0.99) | 0.93 (0.88, 0.99) | 0.97 (0.87, 1.08) | 1.04 (0.92, 1.18) | 0.49 |
| 108 | cg05215649^c^ | NADSYN1 | 0.91 (0.85, 0.97) | 0.89 (0.82, 0.97) | 0.95 (0.81, 1.11) | 1.06 (0.89, 1.26) | 0.49 |
| 109 | cg26044621^c^ | DHCR7 | 1.02 (0.97, 1.07) | 1.04 (0.98, 1.10) | 1.00 (0.90, 1.11) | 0.96 (0.86, 1.08) | 0.50 |
| 110 | cg23654431^c^ | VDR | 1.03 (0.99, 1.06) | 1.02 (0.98, 1.06) | 1.05 (0.98, 1.13) | 1.03 (0.95, 1.12) | 0.50 |
| 111 | cg14022501 | RXRA | 1.03 (0.98, 1.07) | 1.02 (0.97, 1.07) | 1.05 (0.96, 1.16) | 1.04 (0.93, 1.15) | 0.50 |
| 112 | cg00287413 | CYP24A1 | 1.01 (0.99, 1.03) | 1.01 (0.99, 1.04) | 0.99 (0.95, 1.04) | 0.98 (0.93, 1.03) | 0.50 |
| 113 | cg14051721 | RXRA | 1.02 (0.97, 1.07) | 1.00 (0.95, 1.06) | 1.05 (0.94, 1.17) | 1.04 (0.92, 1.18) | 0.51 |
| 114 | cg14570632^c^ | RXRA | 0.92 (0.86, 0.97) | 0.94 (0.88, 1.00) | 0.89 (0.77, 1.03) | 0.95 (0.81, 1.11) | 0.52 |
| 115 | cg25377865 | GC | 0.99 (0.94, 1.03) | 0.99 (0.94, 1.04) | 0.95 (0.86, 1.05) | 0.96 (0.86, 1.08) | 0.52 |
| 116 | cg00545196^c^ | RXRA | 0.99 (0.94, 1.04) | 1.00 (0.95, 1.06) | 0.96 (0.86, 1.07) | 0.96 (0.85, 1.09) | 0.53 |
| 117 | cg20733846^c^ | NADSYN1 | 1.08 (0.99, 1.18) | 1.11 (1.00, 1.23) | 1.03 (0.83, 1.28) | 0.93 (0.73, 1.18) | 0.55 |
| 118 | cg02143877^c^ | CYP24A1 | 1.02 (0.98, 1.06) | 1.03 (0.98, 1.08) | 1.00 (0.93, 1.08) | 0.97 (0.89, 1.07) | 0.56 |
| 119 | cg25623977^c^ | NADSYN1 | 1.01 (0.96, 1.06) | 0.99 (0.94, 1.06) | 1.03 (0.92, 1.16) | 1.04 (0.91, 1.18) | 0.56 |
| 120 | cg03544918^c^ | CYP2R1 | 1.01 (0.97, 1.05) | 1.00 (0.95, 1.05) | 1.03 (0.94, 1.12) | 1.03 (0.93, 1.14) | 0.57 |
| 121 | cg07212292^c^ | NADSYN1 | 1.04 (0.97, 1.12) | 1.06 (0.97, 1.16) | 1.01 (0.86, 1.18) | 0.95 (0.79, 1.14) | 0.57 |
| 122 | cg06326648^c^ | CYP2R1 | 0.97 (0.92, 1.02) | 0.97 (0.92, 1.03) | 0.94 (0.83, 1.05) | 0.96 (0.84, 1.10) | 0.57 |
| 123 | cg14545975 | RXRA | 0.99 (0.94, 1.05) | 0.99 (0.93, 1.06) | 1.03 (0.90, 1.18) | 1.04 (0.90, 1.21) | 0.59 |
| 124 | cg01476789^c^ | DHCR7 | 0.99 (0.94, 1.04) | 0.98 (0.92, 1.04) | 1.01 (0.91, 1.11) | 1.03 (0.92, 1.16) | 0.60 |
| 125 | cg06417365^c^ | NADSYN1 | 0.94 (0.89, 1.00) | 0.94 (0.88, 1.01) | 0.91 (0.80, 1.03) | 0.96 (0.83, 1.11) | 0.60 |
| 126 | cg14654324^c^ | RXRA | 1.01 (0.97, 1.06) | 1.01 (0.96, 1.06) | 1.03 (0.94, 1.13) | 1.03 (0.93, 1.14) | 0.61 |
| 127 | cg05240381^c^ | NADSYN1 | 1.02 (0.97, 1.07) | 1.00 (0.95, 1.06) | 1.03 (0.94, 1.14) | 1.03 (0.92, 1.15) | 0.62 |
| 128 | cg02411493^c^ | NADSYN1 | 0.95 (0.90, 1.00) | 0.94 (0.89, 1.00) | 0.97 (0.87, 1.07) | 1.03 (0.92, 1.16) | 0.62 |
| 129 | cg02604290^c^ | CYP24A1 | 0.99 (0.96, 1.01) | 0.99 (0.96, 1.02) | 0.98 (0.92, 1.03) | 0.98 (0.92, 1.05) | 0.63 |
| 130 | cg02319187^c^ | RXRA | 1.00 (0.95, 1.05) | 0.99 (0.94, 1.05) | 1.02 (0.92, 1.12) | 1.03 (0.92, 1.15) | 0.64 |
| 131 | cg07185038^c^ | CYP27B1 | 1.03 (0.98, 1.08) | 1.03 (0.97, 1.09) | 1.06 (0.95, 1.17) | 1.03 (0.91, 1.16) | 0.64 |
| 132 | cg05163923^c^ | DHCR7 | 0.96 (0.92, 1.01) | 0.95 (0.91, 1.00) | 0.98 (0.89, 1.08) | 1.03 (0.92, 1.14) | 0.65 |
| 133 | cg09800519^c^ | RXRA | 1.05 (1.02, 1.09) | 1.04 (1.01, 1.08) | 1.06 (0.99, 1.14) | 1.02 (0.94, 1.10) | 0.65 |
| 134 | cg12960201^c^ | DHCR7 | 1.02 (0.97, 1.08) | 1.03 (0.96, 1.10) | 1.00 (0.90, 1.11) | 0.97 (0.86, 1.10) | 0.66 |
| 135 | cg25543401^c^ | NADSYN1 | 0.98 (0.92, 1.05) | 0.98 (0.90, 1.06) | 1.01 (0.87, 1.18) | 1.04 (0.87, 1.23) | 0.67 |
| 136 | cg19008693^c^ | VDR | 0.98 (0.94, 1.03) | 0.99 (0.94, 1.05) | 0.97 (0.87, 1.07) | 0.97 (0.87, 1.10) | 0.67 |
| 137 | cg13689699^c^ | RXRA | 0.90 (0.85, 0.95) | 0.89 (0.83, 0.95) | 0.91 (0.81, 1.03) | 1.03 (0.90, 1.18) | 0.68 |
| 138 | cg20372759^c^ | CYP27B1 | 1.03 (0.98, 1.08) | 1.04 (0.97, 1.10) | 1.01 (0.90, 1.13) | 0.97 (0.86, 1.11) | 0.68 |
| 139 | cg04875697^c^ | RXRA | 0.93 (0.89, 0.98) | 0.92 (0.87, 0.97) | 0.95 (0.85, 1.06) | 1.03 (0.91, 1.16) | 0.68 |
| 140 | cg13931640 | RXRA | 0.93 (0.88, 0.99) | 0.92 (0.86, 1.00) | 0.95 (0.85, 1.07) | 1.03 (0.90, 1.18) | 0.69 |
| 141 | cg23792245^c^ | CYP2R1 | 0.97 (0.93, 1.02) | 0.97 (0.92, 1.02) | 0.95 (0.87, 1.04) | 0.98 (0.88, 1.08) | 0.69 |
| 142 | cg05732940^c^ | CYP27B1 | 0.99 (0.96, 1.02) | 0.99 (0.95, 1.03) | 0.98 (0.92, 1.04) | 0.98 (0.91, 1.06) | 0.69 |
| 143 | cg05454237^c^ | CYP2R1 | 1.01 (0.97, 1.05) | 1.00 (0.96, 1.05) | 1.02 (0.94, 1.12) | 1.02 (0.92, 1.13) | 0.69 |
| 144 | cg16910670^c^ | NADSYN1 | 0.99 (0.94, 1.03) | 0.99 (0.94, 1.04) | 1.01 (0.91, 1.12) | 1.02 (0.91, 1.15) | 0.69 |
| 145 | cg06883848^c^ | CYP27B1 | 1.00 (0.97, 1.03) | 1.01 (0.97, 1.04) | 0.99 (0.93, 1.05) | 0.99 (0.92, 1.06) | 0.70 |
| 146 | cg14462321^c^ | RXRA | 1.00 (0.95, 1.06) | 1.00 (0.93, 1.06) | 1.02 (0.91, 1.14) | 1.03 (0.90, 1.17) | 0.71 |
| 147 | cg20006855^c^ | NADSYN1 | 0.96 (0.89, 1.02) | 0.96 (0.88, 1.04) | 0.93 (0.81, 1.07) | 0.97 (0.82, 1.14) | 0.71 |
| 148 | cg04946948^c^ | NADSYN1 | 0.95 (0.90, 1.00) | 0.95 (0.89, 1.01) | 0.97 (0.86, 1.09) | 1.02 (0.89, 1.17) | 0.73 |
| 149 | cg13425135 | RXRA | 1.02 (0.95, 1.09) | 1.04 (0.96, 1.12) | 1.00 (0.85, 1.19) | 0.97 (0.80, 1.17) | 0.73 |
| 150 | cg14484045^c^ | RXRA | 1.01 (0.96, 1.06) | 1.00 (0.94, 1.06) | 1.02 (0.92, 1.13) | 1.02 (0.91, 1.15) | 0.73 |
| 151 | cg01462727 | CYP24A1 | 1.00 (0.98, 1.02) | 0.99 (0.97, 1.02) | 1.00 (0.96, 1.05) | 1.01 (0.96, 1.06) | 0.73 |
| 152 | cg19794395 | CYP27B1 | 0.93 (0.88, 0.98) | 0.92 (0.87, 0.98) | 0.94 (0.85, 1.04) | 1.02 (0.90, 1.15) | 0.74 |
| 153 | cg24229579^c^ | NADSYN1 | 0.98 (0.93, 1.04) | 0.98 (0.91, 1.04) | 1.00 (0.90, 1.10) | 1.02 (0.90, 1.15) | 0.75 |
| 154 | cg21201884^c^ | NADSYN1 | 1.05 (1.00, 1.10) | 1.05 (0.99, 1.11) | 1.03 (0.93, 1.14) | 0.98 (0.87, 1.11) | 0.76 |
| 155 | cg20321331^c^ | CYP2R1 | 0.98 (0.94, 1.01) | 0.98 (0.94, 1.01) | 0.97 (0.90, 1.03) | 0.99 (0.91, 1.07) | 0.76 |
| 156 | cg25219939^c^ | VDR | 1.01 (0.96, 1.05) | 1.01 (0.96, 1.07) | 1.03 (0.92, 1.15) | 1.02 (0.90, 1.15) | 0.77 |
| 157 | cg02673418^c^ | CYP24A1 | 1.02 (0.97, 1.06) | 1.02 (0.97, 1.07) | 1.01 (0.92, 1.10) | 0.99 (0.89, 1.09) | 0.77 |
| 158 | cg14265220^c^ | RXRA | 0.98 (0.94, 1.02) | 0.98 (0.93, 1.02) | 0.99 (0.91, 1.08) | 1.01 (0.92, 1.12) | 0.78 |
| 159 | cg23685712 | CYP24A1 | 1.00 (0.98, 1.03) | 1.01 (0.98, 1.04) | 1.00 (0.93, 1.06) | 0.99 (0.92, 1.06) | 0.78 |
| 160 | cg04837494 | GC | 1.02 (0.98, 1.06) | 1.03 (0.98, 1.08) | 1.04 (0.95, 1.15) | 1.01 (0.91, 1.12) | 0.79 |
| 161 | cg08752080^c^ | GC | 0.98 (0.94, 1.03) | 0.98 (0.92, 1.03) | 0.99 (0.89, 1.11) | 1.02 (0.90, 1.15) | 0.79 |
| 162 | cg11383802^c^ | DHCR7 | 0.96 (0.89, 1.04) | 0.95 (0.86, 1.04) | 0.97 (0.82, 1.15) | 1.03 (0.85, 1.24) | 0.79 |
| 163 | cg02547054 | VDR | 1.00 (0.95, 1.07) | 0.99 (0.93, 1.07) | 1.01 (0.89, 1.15) | 1.02 (0.88, 1.18) | 0.80 |
| 164 | cg10195011^c^ | VDR | 0.91 (0.86, 0.96) | 0.91 (0.85, 0.97) | 0.89 (0.79, 1.01) | 0.98 (0.85, 1.13) | 0.81 |
| 165 | cg13855454 | VDR | 0.99 (0.94, 1.04) | 0.99 (0.94, 1.05) | 0.98 (0.90, 1.07) | 0.99 (0.89, 1.10) | 0.81 |
| 166 | cg02972188^c^ | CYP27B1 | 1.04 (1.01, 1.07) | 1.04 (1.00, 1.07) | 1.03 (0.97, 1.09) | 0.99 (0.93, 1.06) | 0.81 |
| 167 | cg25305530^c^ | CYP24A1 | 1.02 (0.96, 1.09) | 1.03 (0.96, 1.10) | 1.05 (0.90, 1.21) | 1.02 (0.87, 1.20) | 0.83 |
| 168 | cg25454890^c^ | CYP2R1 | 1.01 (0.97, 1.04) | 1.00 (0.96, 1.05) | 1.01 (0.94, 1.09) | 1.01 (0.93, 1.10) | 0.83 |
| 169 | cg03490288^c^ | DHCR7 | 1.00 (0.96, 1.04) | 1.00 (0.96, 1.05) | 0.99 (0.90, 1.09) | 0.99 (0.89, 1.10) | 0.83 |
| 170 | cg03018369^c^ | NADSYN1 | 0.92 (0.86, 0.99) | 0.94 (0.86, 1.01) | 0.92 (0.78, 1.08) | 0.98 (0.82, 1.18) | 0.84 |
| 171 | cg21560157^c^ | NADSYN1 | 0.93 (0.84, 1.02) | 0.92 (0.82, 1.03) | 0.94 (0.78, 1.13) | 1.02 (0.82, 1.27) | 0.85 |
| 172 | cg05896371^c^ | DHCR7 | 1.00 (0.96, 1.05) | 1.00 (0.95, 1.05) | 1.01 (0.91, 1.11) | 1.01 (0.91, 1.13) | 0.85 |
| 173 | cg06169276^c^ | NADSYN1 | 1.06 (0.99, 1.14) | 1.06 (0.97, 1.15) | 1.04 (0.88, 1.23) | 0.98 (0.82, 1.18) | 0.85 |
| 174 | cg11724562^c^ | NADSYN1 | 1.05 (1.00, 1.10) | 1.05 (0.99, 1.11) | 1.06 (0.96, 1.17) | 1.01 (0.90, 1.13) | 0.85 |
| 175 | cg27254468^c^ | NADSYN1 | 0.97 (0.93, 1.02) | 0.97 (0.92, 1.02) | 0.98 (0.89, 1.07) | 1.01 (0.91, 1.12) | 0.86 |
| 176 | cg06523128^c^ | DHCR7 | 0.95 (0.91, 0.99) | 0.94 (0.90, 0.99) | 0.95 (0.87, 1.03) | 1.01 (0.91, 1.11) | 0.88 |
| 177 | cg12090177^c^ | NADSYN1 | 0.93 (0.87, 0.99) | 0.93 (0.86, 1.01) | 0.92 (0.80, 1.06) | 0.99 (0.84, 1.16) | 0.88 |
| 178 | cg24806812 | GC | 1.09 (1.04, 1.14) | 1.09 (1.04, 1.15) | 1.10 (1.01, 1.20) | 1.01 (0.91, 1.11) | 0.89 |
| 179 | cg02220233 | GC | 0.98 (0.95, 1.01) | 0.98 (0.94, 1.02) | 0.97 (0.91, 1.05) | 0.99 (0.92, 1.08) | 0.89 |
| 180 | cg01604138^c^ | NADSYN1 | 1.01 (0.96, 1.06) | 1.00 (0.94, 1.07) | 1.01 (0.90, 1.14) | 1.01 (0.88, 1.16) | 0.90 |
| 181 | cg02087383 | GC | 1.00 (0.96, 1.05) | 1.00 (0.95, 1.05) | 1.01 (0.91, 1.12) | 1.01 (0.90, 1.13) | 0.90 |
| 182 | cg17997279 | CYP24A1 | 0.99 (0.96, 1.01) | 0.99 (0.95, 1.02) | 0.98 (0.92, 1.04) | 1.00 (0.93, 1.07) | 0.90 |
| 183 | cg24582168 | CYP24A1 | 1.00 (0.96, 1.04) | 1.00 (0.95, 1.05) | 0.99 (0.91, 1.08) | 0.99 (0.90, 1.10) | 0.92 |
| 184 | cg27537561^c^ | VDR | 1.06 (0.98, 1.13) | 1.05 (0.97, 1.14) | 1.04 (0.89, 1.22) | 0.99 (0.83, 1.19) | 0.92 |
| 185 | cg10847948^c^ | NADSYN1 | 1.00 (0.96, 1.03) | 1.00 (0.96, 1.04) | 0.99 (0.92, 1.07) | 1.00 (0.92, 1.08) | 0.93 |
| 186 | cg07060721 | CYP27B1 | 1.03 (1.00, 1.06) | 1.03 (1.00, 1.07) | 1.04 (0.97, 1.10) | 1.00 (0.94, 1.07) | 0.94 |
| 187 | cg17773225^c^ | CYP27B1 | 0.98 (0.95, 1.00) | 0.98 (0.95, 1.01) | 0.97 (0.92, 1.03) | 1.00 (0.94, 1.06) | 0.94 |
| 188 | cg27048527 ^c^ | NADSYN1 | 0.97 (0.92, 1.02) | 0.96 (0.91, 1.03) | 0.97 (0.87, 1.09) | 1.00 (0.88, 1.14) | 0.95 |
| 189 | cg00455178^c^ | CYP2R1 | 1.00 (0.95, 1.05) | 1.00 (0.94, 1.06) | 1.00 (0.89, 1.12) | 1.00 (0.88, 1.15) | 0.95 |
| 190 | cg09816180^c^ | GC | 1.02 (0.96, 1.08) | 1.02 (0.96, 1.10) | 1.03 (0.92, 1.15) | 1.00 (0.88, 1.14) | 0.95 |
| 191 | cg21843272 | VDR | 1.01 (0.98, 1.04) | 1.01 (0.97, 1.05) | 1.01 (0.94, 1.08) | 1.00 (0.92, 1.08) | 0.95 |
| 192 | cg00268518^c^ | NADSYN1 | 0.96 (0.91, 1.01) | 0.96 (0.90, 1.02) | 0.96 (0.86, 1.07) | 1.00 (0.88, 1.14) | 0.95 |
| 193 | cg00324108^c^ | GC | 0.95 (0.90, 0.99) | 0.95 (0.90, 1.00) | 0.94 (0.86, 1.03) | 1.00 (0.90, 1.11) | 0.96 |
| 194 | cg24826892^c^ | DHCR7 | 0.90 (0.84, 0.97) | 0.90 (0.84, 0.98) | 0.90 (0.77, 1.05) | 1.00 (0.84, 1.18) | 0.96 |
| 195 | cg13847322 | RXRA | 1.02 (0.98, 1.06) | 1.02 (0.97, 1.07) | 1.02 (0.93, 1.12) | 1.00 (0.90, 1.11) | 0.97 |
| 196 | cg13005613^c^ | RXRA | 0.97 (0.92, 1.02) | 0.97 (0.91, 1.03) | 0.97 (0.87, 1.08) | 1.00 (0.88, 1.14) | 0.98 |
| 197 | cg11980188^c^ | CYP2R1 | 1.02 (0.96, 1.08) | 1.02 (0.95, 1.08) | 1.02 (0.91, 1.14) | 1.00 (0.88, 1.14) | 0.99 |
| 198 | cg13487983 | RXRA | 1.00 (0.94, 1.05) | 1.00 (0.93, 1.07) | 1.00 (0.90, 1.11) | 1.00 (0.88, 1.13) | 1.00 |

^a^After excluding those with missing covariate information

^b^Change in the methylation-breast cancer association for being in the 4^th^ quartile of 25(OH)D (>38.0 ng/mL) versus the first three (<38.0 ng/mL). A value >1.00 indicates that the estimated HR for the methylation-breast cancer association is higher among those with higher 25(OH)D levels. Similarly, an RHR <1.00 indicates that the estimated HR for the methylation-breast cancer association is higher among those with lower 25(OH)D levels.

^c^Intraclass correlation coefficient for probe <0.5

**Table S4. Interacting effects of 25(OH)D and CpG sites in vitamin D-related genes on the 5-year risk of post-menopausal breast cancer (852 cases, 1026 from subcohort, including 41 additional cases^a^): Ratio of hazard ratios and 95% confidence intervals**

| **Rank** | **Rank for all breast cancer** | **CpG site** | **Gene** | **Ratio of Hazard Ratios**  **(95% CI)^b^** | **p-value** |
| --- | --- | --- | --- | --- | --- |
| 1 | 2 | cg13786567 | RXRA | 1.52 (1.21, 1.91) | 4.0 x 10^-4^ |
| 2 | 1 | cg21201924 | RXRA | 1.19 (1.07, 1.33) | 0.001 |
| 3 | 5 | cg14154547^c^ | RXRA | 1.35 (1.12, 1.63) | 0.002 |
| 4 | 4 | cg12978433 | CYP24A1 | 0.89 (0.83, 0.96) | 0.002 |
| 5 | 3 | cg02127980 | RXRA | 1.27 (1.07, 1.52) | 0.007 |
| 6 | 15 | cg10592901 | VDR | 0.89 (0.81, 0.97) | 0.008 |
| 7 | 7 | cg13510651^c^ | RXRA | 1.24 (1.04, 1.48) | 0.02 |
| 8 | 13 | cg14854850 | VDR | 0.86 (0.75, 0.97) | 0.02 |
| 9 | 6 | cg18956481^c^ | CYP24A1 | 0.92 (0.86, 0.99) | 0.03 |
| 10 | 23 | cg01630362^c^ | NADSYN1 | 0.88 (0.79, 0.99) | 0.04 |
| 11 | 24 | cg17559402^c^ | NADSYN1 | 1.11 (1.01, 1.23) | 0.04 |
| 12 | 9 | cg09253762 | CYP27B1 | 1.14 (1.00, 1.29) | 0.04 |
| 13 | 12 | cg11035813^c^ | DHCR7 | 1.13 (1.00, 1.27) | 0.05 |
| 14 | 30 | cg13043300^c^ | DHCR7 | 1.12 (0.99, 1.26) | 0.07 |
| 15 | 93 | cg13865488 | RXRA | 0.91 (0.82, 1.01) | 0.08 |
| 16 | 49 | cg16171723^c^ | DHCR7 | 1.15 (0.98, 1.34) | 0.09 |
| 17 | 54 | cg08460032^c^ | CYP2R1 | 1.13 (0.98, 1.31) | 0.09 |
| 18 | 90 | cg17553080^c^ | NADSYN1 | 1.09 (0.99, 1.21) | 0.09 |
| 19 | 14 | cg25588697^c^ | DHCR7 | 1.15 (0.97, 1.37) | 0.10 |
| 20 | 8 | cg14236758 | RXRA | 1.16 (0.97, 1.40) | 0.10 |
| 21 | 39 | cg03284291^c^ | NADSYN1 | 1.10 (0.97, 1.25) | 0.13 |
| 22 | 16 | cg12474705^c^ | NADSYN1 | 0.91 (0.80, 1.03) | 0.13 |
| 23 | 37 | cg16151558^c^ | DHCR7 | 0.92 (0.82, 1.03) | 0.14 |
| 24 | 57 | cg24722099 | CYP27B1 | 0.88 (0.74, 1.04) | 0.14 |
| 25 | 120 | cg03544918^c^ | CYP2R1 | 1.08 (0.97, 1.21) | 0.14 |
| 26 | 11 | cg05072492^c^ | NADSYN1 | 1.11 (0.96, 1.28) | 0.14 |
| 27 | 137 | cg13689699^c^ | RXRA | 1.12 (0.96, 1.31) | 0.15 |
| 28 | 22 | cg14121282 | RXRA | 1.08 (0.97, 1.21) | 0.16 |
| 29 | 107 | cg07099121^c^ | DHCR7 | 1.10 (0.96, 1.27) | 0.17 |
| 30 | 66 | cg13061648^c^ | RXRA | 0.91 (0.79, 1.04) | 0.17 |
| 31 | 21 | cg00753924 | RXRA | 1.03 (0.98, 1.09) | 0.18 |
| 32 | 69 | cg06369854^c^ | VDR | 1.07 (0.97, 1.18) | 0.18 |
| 33 | 48 | cg04321714 | CYP27B1 | 0.95 (0.88, 1.03) | 0.18 |
| 34 | 10 | cg18482822^c^ | DHCR7 | 1.09 (0.96, 1.23) | 0.19 |
| 35 | 51 | cg14204281^c^ | RXRA | 0.94 (0.85, 1.03) | 0.19 |
| 36 | 92 | cg24110768^c^ | CYP27B1 | 1.07 (0.97, 1.17) | 0.19 |
| 37 | 31 | cg27263778^c^ | NADSYN1 | 0.91 (0.79, 1.05) | 0.20 |
| 38 | 46 | cg15757022^c^ | NADSYN1 | 0.89 (0.75, 1.06) | 0.20 |
| 39 | 29 | cg07487535^c^ | DHCR7 | 1.07 (0.97, 1.18) | 0.20 |
| 40 | 27 | cg13413384 | RXRA | 1.15 (0.93, 1.43) | 0.20 |
| 41 | 25 | cg05785753 | NADSYN1 | 1.07 (0.96, 1.19) | 0.21 |
| 42 | 95 | cg03307911^c^ | CYP24A1 | 1.09 (0.95, 1.26) | 0.22 |
| 43 | 28 | cg07376029 | GC | 1.06 (0.97, 1.17) | 0.22 |
| 44 | 132 | cg05163923^c^ | DHCR7 | 1.08 (0.96, 1.22) | 0.22 |
| 45 | 19 | cg23101118 | CYP27B1 | 1.05 (0.97, 1.12) | 0.22 |
| 46 | 56 | cg24849555^c^ | CYP24A1 | 1.07 (0.96, 1.20) | 0.23 |
| 47 | 47 | cg14311020^c^ | VDR | 1.12 (0.93, 1.34) | 0.23 |
| 48 | 36 | cg05220083^c^ | CYP27B1 | 1.11 (0.94, 1.31) | 0.23 |
| 49 | 35 | cg19227606^c^ | NADSYN1 | 1.09 (0.95, 1.26) | 0.23 |
| 50 | 41 | cg14468605^c^ | RXRA | 0.88 (0.72, 1.08) | 0.23 |
| 51 | 38 | cg09290506^c^ | CYP27B1 | 1.14 (0.92, 1.41) | 0.23 |
| 52 | 167 | cg25305530^c^ | CYP24A1 | 1.12 (0.93, 1.34) | 0.24 |
| 53 | 63 | cg14051662^c^ | RXRA | 1.08 (0.94, 1.25) | 0.25 |
| 54 | 26 | cg02470587^c^ | VDR | 1.07 (0.95, 1.20) | 0.25 |
| 55 | 17 | cg16984335^c^ | CYP27B1 | 0.94 (0.84, 1.05) | 0.26 |
| 56 | 81 | cg19089328 | NADSYN1 | 1.04 (0.97, 1.12) | 0.27 |
| 57 | 166 | cg02972188^c^ | CYP27B1 | 0.96 (0.89, 1.03) | 0.27 |
| 58 | 177 | cg12090177^c^ | NADSYN1 | 0.91 (0.76, 1.08) | 0.27 |
| 59 | 172 | cg05896371^c^ | DHCR7 | 0.93 (0.82, 1.06) | 0.28 |
| 60 | 106 | cg24164254^c^ | RXRA | 1.11 (0.92, 1.35) | 0.28 |
| 61 | 33 | cg23305502^c^ | NADSYN1 | 0.88 (0.70, 1.11) | 0.29 |
| 62 | 151 | cg01462727 | CYP24A1 | 1.03 (0.98, 1.08) | 0.29 |
| 63 | 86 | cg02399233^c^ | NADSYN1 | 0.87 (0.67, 1.13) | 0.30 |
| 64 | 20 | cg19345671^c^ | NADSYN1 | 0.90 (0.73, 1.11) | 0.31 |
| 65 | 73 | cg13936846^c^ | RXRA | 1.06 (0.95, 1.19) | 0.31 |
| 66 | 182 | cg17997279 | CYP24A1 | 1.04 (0.96, 1.12) | 0.31 |
| 67 | 18 | cg13941235 | RXRA | 1.04 (0.97, 1.11) | 0.31 |
| 68 | 122 | cg06326648^c^ | CYP2R1 | 0.92 (0.77, 1.09) | 0.33 |
| 69 | 59 | cg07617163^c^ | NADSYN1 | 1.09 (0.92, 1.28) | 0.33 |
| 70 | 82 | cg16544887^c^ | CYP2R1 | 0.94 (0.83, 1.06) | 0.34 |
| 71 | 181 | cg02087383 | GC | 1.06 (0.94, 1.21) | 0.35 |
| 72 | 52 | cg06146607^c^ | NADSYN1 | 1.07 (0.93, 1.24) | 0.35 |
| 73 | 45 | cg02059519 | RXRA | 1.08 (0.92, 1.26) | 0.35 |
| 74 | 40 | cg03146219 | NADSYN1 | 1.04 (0.96, 1.13) | 0.36 |
| 75 | 74 | cg01182309 | CYP27B1 | 0.98 (0.95, 1.02) | 0.37 |
| 76 | 70 | cg13978325^c^ | RXRA | 1.03 (0.96, 1.11) | 0.38 |
| 77 | 32 | cg09997530 | GC | 0.94 (0.83, 1.07) | 0.38 |
| 78 | 44 | cg01065422^c^ | NADSYN1 | 1.06 (0.93, 1.21) | 0.39 |
| 79 | 55 | cg04329455^c^ | RXRA | 0.95 (0.84, 1.07) | 0.39 |
| 80 | 72 | cg08581512^c^ | CYP2R1 | 0.93 (0.79, 1.10) | 0.39 |
| 81 | 141 | cg23792245^c^ | CYP2R1 | 0.95 (0.85, 1.06) | 0.39 |
| 82 | 64 | cg08854599^c^ | NADSYN1 | 0.93 (0.78, 1.10) | 0.40 |
| 83 | 68 | cg16802027^c^ | CYP2R1 | 0.95 (0.85, 1.06) | 0.40 |
| 84 | 79 | cg18413900 | CYP27B1 | 1.08 (0.90, 1.28) | 0.40 |
| 85 | 99 | cg24341498^c^ | RXRA | 1.02 (0.98, 1.05) | 0.41 |
| 86 | 175 | cg27254468^c^ | NADSYN1 | 1.05 (0.93, 1.18) | 0.41 |
| 87 | 43 | cg04905829 | CYP27B1 | 0.94 (0.82, 1.09) | 0.41 |
| 88 | 124 | cg01476789^c^ | DHCR7 | 1.05 (0.93, 1.20) | 0.42 |
| 89 | 142 | cg05732940^c^ | CYP27B1 | 0.97 (0.89, 1.05) | 0.42 |
| 90 | 60 | cg00630245^c^ | DHCR7 | 0.94 (0.81, 1.09) | 0.42 |
| 91 | 138 | cg20372759^c^ | CYP27B1 | 0.94 (0.82, 1.09) | 0.43 |
| 92 | 194 | cg24826892^c^ | DHCR7 | 1.08 (0.89, 1.30) | 0.43 |
| 93 | 198 | cg13487983 | RXRA | 0.95 (0.83, 1.09) | 0.44 |
| 94 | 113 | cg14051721 | RXRA | 1.05 (0.92, 1.20) | 0.44 |
| 95 | 133 | cg09800519^c^ | RXRA | 1.04 (0.95, 1.13) | 0.44 |
| 96 | 84 | cg16408394^c^ | RXRA | 0.96 (0.88, 1.06) | 0.44 |
| 97 | 165 | cg13855454 | NA | 0.96 (0.85, 1.07) | 0.45 |
| 98 | 128 | cg02411493^c^ | NADSYN1 | 1.06 (0.92, 1.21) | 0.45 |
| 99 | 58 | cg03137447^c^ | VDR | 1.04 (0.93, 1.17) | 0.46 |
| 100 | 89 | cg03589557^c^ | NADSYN1 | 0.96 (0.86, 1.07) | 0.46 |
| 101 | 88 | cg07864189^c^ | NADSYN1 | 0.96 (0.86, 1.07) | 0.46 |
| 102 | 61 | cg02712555^c^ | CYP24A1 | 1.05 (0.93, 1.18) | 0.46 |
| 103 | 127 | cg05240381^c^ | NADSYN1 | 1.05 (0.93, 1.18) | 0.46 |
| 104 | 116 | cg00545196 ^c^ | RXRA | 0.95 (0.83, 1.09) | 0.47 |
| 105 | 65 | cg12751354 | CYP24A1 | 0.96 (0.87, 1.07) | 0.48 |
| 106 | 104 | cg13677043 | RXRA | 1.05 (0.91, 1.22) | 0.48 |
| 107 | 78 | cg07793224^c^ | NADSYN1 | 0.96 (0.86, 1.07) | 0.48 |
| 108 | 97 | cg13595495^c^ | RXRA | 1.05 (0.92, 1.19) | 0.49 |
| 109 | 76 | cg16321474^c^ | VDR | 0.98 (0.91, 1.05) | 0.49 |
| 110 | 105 | cg10763288 | DHCR7 | 1.07 (0.89, 1.28) | 0.49 |
| 111 | 62 | cg01892327^c^ | DHCR7 | 0.96 (0.85, 1.08) | 0.49 |
| 112 | 118 | cg02143877^c^ | CYP24A1 | 0.96 (0.87, 1.07) | 0.49 |
| 113 | 173 | cg06169276^c^ | NADSYN1 | 1.07 (0.87, 1.33) | 0.50 |
| 114 | 114 | cg14570632^c^ | RXRA | 0.94 (0.79, 1.12) | 0.51 |
| 115 | 77 | cg10037049 | VDR | 0.98 (0.92, 1.04) | 0.51 |
| 116 | 103 | cg05963690^c^ | NADSYN1 | 0.96 (0.84, 1.09) | 0.51 |
| 117 | 67 | cg14472716 | RXRA | 0.95 (0.83, 1.10) | 0.51 |
| 118 | 71 | cg22960616^c^ | NADSYN1 | 1.04 (0.91, 1.19) | 0.53 |
| 119 | 50 | cg04774822 | NADSYN1 | 1.05 (0.89, 1.24) | 0.53 |
| 120 | 176 | cg06523128^c^ | DHCR7 | 1.03 (0.93, 1.15) | 0.55 |
| 121 | 148 | cg04946948^c^ | NADSYN1 | 1.05 (0.90, 1.23) | 0.55 |
| 122 | 98 | cg14344989^c^ | RXRA | 0.97 (0.86, 1.08) | 0.56 |
| 123 | 34 | cg13746518 | RXRA | 1.02 (0.95, 1.10) | 0.57 |
| 124 | 96 | cg26528620^c^ | CYP2R1 | 0.96 (0.82, 1.12) | 0.57 |
| 125 | 80 | cg13556224 | VDR | 0.98 (0.90, 1.06) | 0.57 |
| 126 | 168 | cg25454890^c^ | CYP2R1 | 0.98 (0.89, 1.07) | 0.61 |
| 127 | 197 | cg11980188^c^ | CYP2R1 | 1.04 (0.90, 1.20) | 0.61 |
| 128 | 129 | cg02604290^c^ | CYP24A1 | 0.98 (0.91, 1.05) | 0.61 |
| 129 | 174 | cg11724562^c^ | NADSYN1 | 1.03 (0.91, 1.17) | 0.62 |
| 130 | 185 | cg10847948^c^ | NADSYN1 | 1.02 (0.93, 1.12) | 0.62 |
| 131 | 85 | cg13687497 | RXRA | 1.05 (0.88, 1.25) | 0.62 |
| 132 | 91 | cg18210221^c^ | DHCR7 | 1.06 (0.82, 1.38) | 0.63 |
| 133 | 110 | cg23654431^c^ | VDR | 1.02 (0.94, 1.12) | 0.63 |
| 134 | 163 | cg02547054 | VDR | 1.04 (0.88, 1.22) | 0.64 |
| 135 | 162 | cg11383802 ^c^ | DHCR7 | 0.95 (0.77, 1.18) | 0.65 |
| 136 | 190 | cg09816180^c^ | GC | 0.97 (0.83, 1.12) | 0.66 |
| 137 | 134 | cg12960201^c^ | DHCR7 | 0.97 (0.84, 1.12) | 0.66 |
| 138 | 75 | cg11227966^c^ | CYP2R1 | 0.97 (0.83, 1.13) | 0.67 |
| 139 | 117 | cg20733846^c^ | NADSYN1 | 0.94 (0.73, 1.23) | 0.67 |
| 140 | 121 | cg07212292^c^ | NADSYN1 | 0.96 (0.78, 1.17) | 0.68 |
| 141 | 131 | cg07185038^c^ | CYP27B1 | 1.03 (0.90, 1.17) | 0.68 |
| 142 | 42 | cg04460185^c^ | DHCR7 | 1.02 (0.92, 1.14) | 0.68 |
| 143 | 193 | cg00324108^c^ | GC | 0.98 (0.87, 1.10) | 0.69 |
| 144 | 196 | cg13005613^c^ | RXRA | 1.03 (0.89, 1.18) | 0.69 |
| 145 | 154 | cg21201884^c^ | NADSYN1 | 0.97 (0.86, 1.11) | 0.69 |
| 146 | 152 | cg19794395 | CYP27B1 | 1.03 (0.90, 1.17) | 0.69 |
| 147 | 189 | cg00455178^c^ | CYP2R1 | 0.97 (0.84, 1.13) | 0.70 |
| 148 | 147 | cg20006855^c^ | NADSYN1 | 0.96 (0.80, 1.16) | 0.70 |
| 149 | 109 | cg26044621^c^ | DHCR7 | 0.98 (0.86, 1.11) | 0.71 |
| 150 | 156 | cg25219939^c^ | VDR | 1.03 (0.89, 1.18) | 0.71 |
| 151 | 100 | cg13865595^c^ | VDR | 0.98 (0.87, 1.10) | 0.72 |
| 152 | 144 | cg16910670^c^ | NADSYN1 | 0.98 (0.86, 1.11) | 0.72 |
| 153 | 94 | cg01385795^c^ | CYP2R1 | 0.99 (0.91, 1.07) | 0.72 |
| 154 | 140 | cg13931640 | RXRA | 1.03 (0.88, 1.20) | 0.73 |
| 155 | 153 | cg24229579^c^ | NADSYN1 | 1.03 (0.89, 1.18) | 0.73 |
| 156 | 53 | cg11597185^c^ | DHCR7 | 1.02 (0.91, 1.14) | 0.73 |
| 157 | 195 | cg13847322 | RXRA | 1.02 (0.91, 1.14) | 0.73 |
| 158 | 161 | cg08752080^c^ | GC | 1.02 (0.89, 1.18) | 0.73 |
| 159 | 101 | cg18313719^c^ | NADSYN1 | 1.02 (0.91, 1.15) | 0.73 |
| 160 | 164 | cg10195011^c^ | VDR | 1.03 (0.88, 1.20) | 0.75 |
| 161 | 126 | cg14654324^c^ | RXRA | 1.02 (0.91, 1.14) | 0.75 |
| 162 | 87 | cg02121892^c^ | NADSYN1 | 0.98 (0.85, 1.12) | 0.75 |
| 163 | 139 | cg04875697^c^ | RXRA | 1.02 (0.89, 1.17) | 0.76 |
| 164 | 159 | cg23685712 | CYP24A1 | 1.01 (0.94, 1.09) | 0.76 |
| 165 | 186 | cg07060721 | CYP27B1 | 0.99 (0.92, 1.07) | 0.77 |
| 166 | 119 | cg25623977^c^ | NADSYN1 | 0.98 (0.84, 1.14) | 0.77 |
| 167 | 149 | cg13425135 | RXRA | 0.97 (0.79, 1.19) | 0.78 |
| 168 | 108 | cg05215649^c^ | NADSYN1 | 1.03 (0.85, 1.24) | 0.79 |
| 169 | 143 | cg05454237^c^ | CYP2R1 | 1.02 (0.91, 1.14) | 0.80 |
| 170 | 192 | cg00268518^c^ | NADSYN1 | 1.02 (0.88, 1.17) | 0.80 |
| 171 | 125 | cg06417365 ^c^ | NADSYN1 | 0.98 (0.82, 1.17) | 0.80 |
| 172 | 178 | cg24806812 | GC | 0.99 (0.88, 1.10) | 0.81 |
| 173 | 171 | cg21560157^c^ | NADSYN1 | 0.97 (0.77, 1.23) | 0.81 |
| 174 | 123 | cg14545975 | RXRA | 1.02 (0.87, 1.20) | 0.81 |
| 175 | 158 | cg14265220^c^ | RXRA | 0.99 (0.88, 1.10) | 0.82 |
| 176 | 150 | cg14484045^c^ | RXRA | 1.02 (0.89, 1.16) | 0.82 |
| 177 | 170 | cg03018369^c^ | NADSYN1 | 1.02 (0.83, 1.26) | 0.82 |
| 178 | 169 | cg03490288^c^ | DHCR7 | 1.01 (0.90, 1.14) | 0.82 |
| 179 | 102 | cg02622885^c^ | CYP2R1 | 0.99 (0.87, 1.12) | 0.86 |
| 180 | 180 | cg01604138^c^ | NADSYN1 | 1.01 (0.87, 1.18) | 0.86 |
| 181 | 146 | cg14462321^c^ | RXRA | 0.99 (0.84, 1.15) | 0.87 |
| 182 | 115 | cg25377865 | GC | 1.01 (0.89, 1.15) | 0.88 |
| 183 | 191 | cg21843272 | VDR | 1.01 (0.92, 1.10) | 0.89 |
| 184 | 160 | cg04837494 | GC | 0.99 (0.88, 1.11) | 0.90 |
| 185 | 187 | cg17773225^c^ | CYP27B1 | 1.00 (0.94, 1.08) | 0.90 |
| 186 | 157 | cg02673418^c^ | CYP24A1 | 1.01 (0.90, 1.12) | 0.90 |
| 187 | 111 | cg14022501 | RXRA | 1.01 (0.90, 1.13) | 0.90 |
| 188 | 130 | cg02319187^c^ | RXRA | 1.01 (0.89, 1.14) | 0.91 |
| 189 | 112 | cg00287413 | CYP24A1 | 1.00 (0.94, 1.05) | 0.92 |
| 190 | 179 | cg02220233 | GC | 1.00 (0.91, 1.09) | 0.93 |
| 191 | 188 | cg27048527^c^ | NADSYN1 | 0.99 (0.86, 1.15) | 0.93 |
| 192 | 83 | cg18787440^c^ | CYP27B1 | 0.99 (0.87, 1.14) | 0.94 |
| 193 | 136 | cg19008693^c^ | VDR | 1.00 (0.88, 1.15) | 0.94 |
| 194 | 184 | cg27537561^c^ | VDR | 0.99 (0.81, 1.22) | 0.95 |
| 195 | 155 | cg20321331^c^ | CYP2R1 | 1.00 (0.92, 1.09) | 0.95 |
| 196 | 183 | cg24582168 | CYP24A1 | 1.00 (0.90, 1.12) | 0.98 |
| 197 | 145 | cg06883848^c^ | CYP27B1 | 1.00 (0.92, 1.08) | 0.98 |
| 198 | 135 | cg25543401^c^ | NADSYN1 | 1.00 (0.83, 1.21) | 0.99 |

^a^After excluding those with missing covariate information

^b^Change in the methylation-breast cancer association for being in the 4^th^ quartile of 25(OH)D (>38.0 ng/mL) versus the first three (<38.0 ng/mL). A value >1.00 indicates that the estimated HR for the methylation-breast cancer association is higher among those with higher 25(OH)D levels. Similarly, an RHR <1.00 indicates that the estimated HR for the methylation-breast cancer association is higher among those with lower 25(OH)D levels.

^c^Intraclass correlation coefficient for probe <0.5

**Table S5. Ratio of hazard ratios and 95% confidence intervals for the interaction between 25(OH)D and methylation on the 5-year risk of post-menopausal breast cancer (852 cases, 1026 from subcohort, including 41 additional cases^a^); CpGs with Fisher combined p-values <1x10^-5^ for subcohort versus case comparison**

| **Rank** | **Rank for all breast cancer** | **CpG site** | **Gene** | **Ratio of Hazard Ratios**  **(95% CI)^b^** | **p-value** |
| --- | --- | --- | --- | --- | --- |
| 1 | 1 | cg08092930 | PPFIA1 | 1.12 (1.03, 1.22) | 0.008 |
| 2 | 4 | cg15544721 | PPP1R9A | 0.87 (0.78, 0.97) | 0.01 |
| 3 | 2 | cg23761815 | SLC29A3 | 1.18 (1.03, 1.36) | 0.02 |
| 4 | 3 | cg13243168 | SMARCD2 | 1.25 (1.04, 1.51) | 0.02 |
| 5 | 9 | cg24350360^c^ | EPHX1 | 1.10 (1.02, 1.20) | 0.02 |
| 6 | 11 | cg10401362 | DNAJB6 | 1.15 (1.02, 1.30) | 0.03 |
| 7 | 5 | cg11568290 | 5p15.1 | 1.21 (1.01, 1.44) | 0.04 |
| 8 | 10 | cg22488164 | PLBD1 | 0.91 (0.82, 1.00) | 0.04 |
| 9 | 7 | cg23839180 | FAM49A | 0.94 (0.87, 1.00) | 0.07 |
| 10 | 6 | cg19420720 | P4HB | 1.20 (0.98, 1.47) | 0.08 |
| 11 | 8 | cg15320474^c^ | UBD | 0.90 (0.79, 1.03) | 0.12 |
| 12 | 12 | cg06177555 | SPN | 1.08 (0.93, 1.24) | 0.33 |
| 13 | 16 | cg23999318 | HIPK2 | 0.95 (0.82, 1.09) | 0.47 |
| 14 | 14 | cg21527411 | GLYAT | 0.97 (0.84, 1.12) | 0.67 |
| 15 | 13 | cg11277126 | TRPC4AP | 1.02 (0.86, 1.20) | 0.82 |
| 16 | 15 | cg09914444 | DMBX1 | 1.00 (0.90, 1.11) | 0.98 |

^a^After excluding those with missing covariate information

^b^Change in the methylation-breast cancer association for being in the 4^th^ quartile of 25(OH)D (>38.0 ng/mL) versus the first three (<38.0 ng/mL). A value >1.00 indicates that the estimated HR for the methylation-breast cancer association is higher among those with higher 25(OH)D levels. Similarly, an RHR <1.00 indicates that the estimated HR for the methylation-breast cancer association is higher among those with lower 25(OH)D levels.

^c^Intraclass correlation coefficient for probe <0.5

**Figure S1. Quantile-quantile plot for the association between the epigenetic-by-25(OH)D interaction term and breast cancer risk among post-menopausal women**


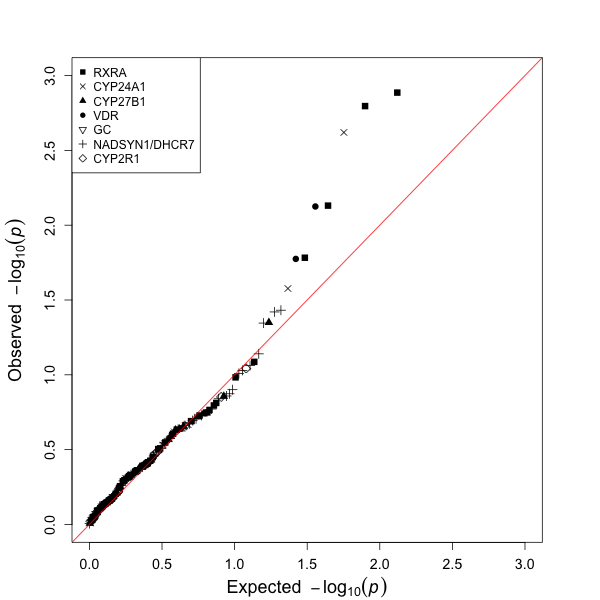


**Figure S2. Volcano plot for the associations between serum 25(OH)D levels (modeled continuously) and DNA methylation at 423,500 CpG sites among 1270 non-Hispanic white women randomly selected from the Sister Study cohort (2003-2009).**

**
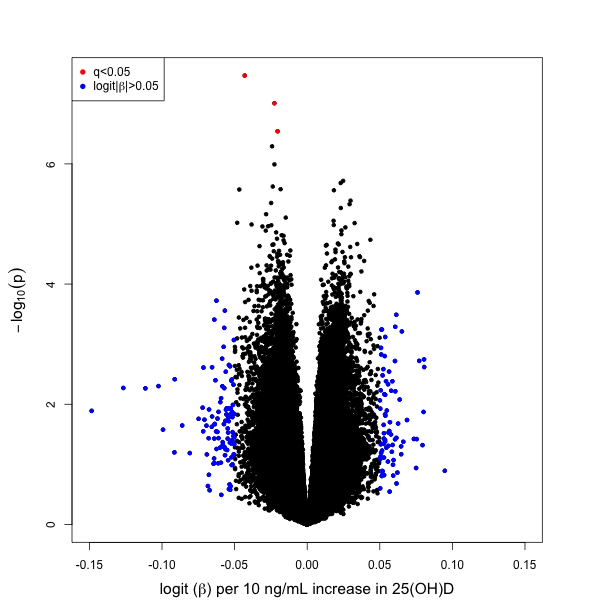
**

**Figure S3.** Manhattan plot (top) and quantile-quantile plot (bottom) for the association between DNA methylation at 423,500 CpG sites and serum 25(OH)D among non-Hispanic white women with breast cancer (n=1024; excluding those who were selected as part of subcohort). No CpGs were statistically significant at q<0.05.


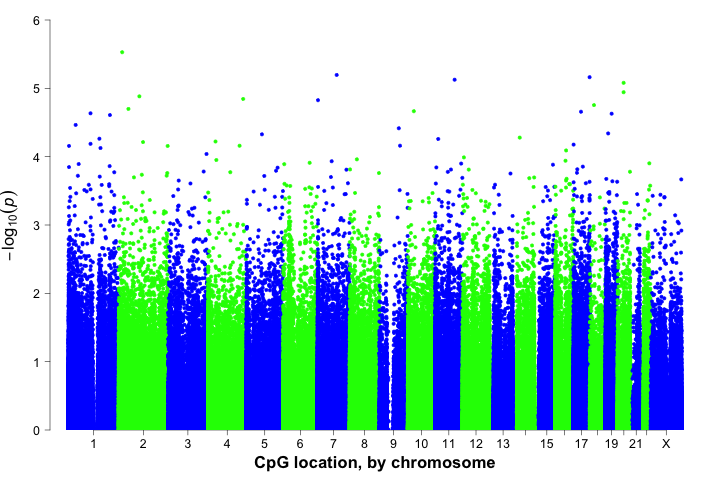


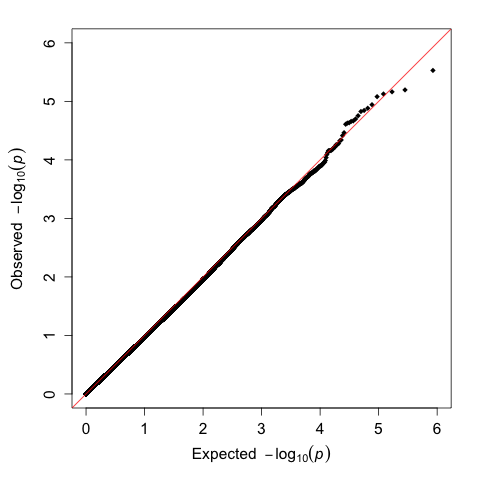

Supplement: Supplementary file 1 — Table S1. Characteristics of participants included in the vitamin D and methylation substudy (Sister Study, 2003–2009); only non-Hispanic white women included. Table S2. Associations between 25(OH)D and methylation at CpG sites in vitamin D-related genes (p > 0.05); Sister Study subcohort (n = 1270). Table S3. Interaction effects of 25(OH)D and methylation at CpG sites in vitamin D-related genes on the 5-year risk of breast cancer (1024 cases, 1270 from subcohort, including 46 additional cases): ratio of hazard ratios and 95% confidence intervals for CpGs with interaction p values > 0.05. Table S4. Interacting effects of 25(OH)D and CpG sites in vitamin D-related genes on the 5-year risk of post-menopausal breast cancer (852 cases, 1026 from subcohort, including 41 additional cases): ratio of hazard ratios and 95% confidence intervals. Table S5. Ratio of hazard ratios and 95% confidence intervals for the interaction between 25(OH)D and methylation on the 5-year risk of postmenopausal breast cancer (852 cases, 1026 from subcohort, including 41 additional cases); CpGs with Fisher combined p values < 1 × 10^−5^ for subcohort versus case comparison. Figure S1. Quantile-quantile plot for the association between the epigenetic-by-25(OH)D interaction term and breast cancer risk among postmenopausal women. Figure S2. Volcano plot for the associations between serum 25(OH)D levels (modeled continuously) and DNA methylation at 423,500 CpG sites among 1270 non-Hispanic white women randomly selected from the Sister Study cohort (2003–2009). Figure S3. Manhattan plot (top) and quantile-quantile plot (bottom) for the association between DNA methylation at 423,500 CpG sites and serum 25(OH)D among non-Hispanic white women with breast cancer (n = 1024; excluding those who were selected as part of subcohort). No CpGs were statistically significant at q < 0.05. (DOCX 419 kb) [file 13058_2018_994_MOESM1_ESM.docx]
